# Relationship of Serum Progesterone and Progesterone Metabolites with Mammographic Breast Density and Terminal Ductal Lobular Unit Involution among Women Undergoing Diagnostic Breast Biopsy

**DOI:** 10.3390/jcm9010245

**Published:** 2020-01-17

**Authors:** Manila Hada, Hannah Oh, Shaoqi Fan, Roni T. Falk, Berta Geller, Pamela Vacek, Donald Weaver, John Shepherd, Jeff Wang, Bo Fan, Sally Herschorn, Louise A. Brinton, Xia Xu, Mark E. Sherman, Britton Trabert, Gretchen L. Gierach

**Affiliations:** 1 Division of Cancer Epidemiology and Genetics, National Cancer Institute, National Institutes of Health, Bethesda, MD 20892, USA; shaoqi.fan@nih.gov (S.F.); falkr@exchange.nih.gov (R.T.F.); brintonl@exchange.nih.gov (L.A.B.); britton.trabert@nih.gov (B.T.); gierachg@mail.nih.gov (G.L.G.); 2 Division of Health Policy and Management, College of Health Science, Korea University, Seoul 02841, Korea; hannahoh@korea.ac.kr; 3 Department of Family Medicine, University of Vermont and Vermont Cancer Center, Burlington, VT 05401, USA; berta.geller@uvm.edu; 4 Department of Medical Biostatistics, University of Vermont and Vermont Cancer Center, Burlington, VT 05401, USA; Pamela.Vacek@uvm.edu; 5 Department of Pathology, University of Vermont and Vermont Cancer Center, Burlington, VT 05401, USA; Donald.Weaver@uvmhealth.org; 6 Population Sciences in the Pacific Program (Cancer Epidemiology), University of Hawaii Cancer Center, Honolulu, HI 96813, USA; johnshep@hawaii.edu; 7 Graduate School of Medicine, Hokkaido University, Sapporo 060-080, Japan; jeff.wing.wang.walla.walla@gmail.com; 8 Department of Radiology & Biomedical Imaging, University of California, San Francisco, CA 94115, USA; Bo.Fan@ucsf.edu; 9 Department of Radiology, University of Vermont and Vermont Cancer Center, Burlington, VT 05401, USA; Sally.Herschorn@uvmhealth.org; 10 Frederick National Laboratory for Cancer Research, Frederick, MD 21701, USA; xuxi@mail.nih.gov; 11 Mayo Clinic, Jacksonville, FL 32224, USA; Sherman.Mark@mayo.edu

**Keywords:** mammographic density, breast density, progesterone, serum progesterone assay, breast cancer, liquid chromatography-tandem mass spectrometry

## Abstract

The association of progesterone/progesterone metabolites with elevated mammographic breast density (MBD) and delayed age-related terminal duct lobular unit (TDLU) involution, strong breast cancer risk factors, has received limited attention. Using a reliable liquid chromatography-tandem mass-spectrometry assay, we quantified serum progesterone/progesterone metabolites and explored cross-sectional relationships with MBD and TDLU involution among women, ages 40–65, undergoing diagnostic breast biopsy. Quantitative MBD measures were estimated in pre-biopsy digital mammograms. TDLU involution was quantified in diagnostic biopsies. Adjusted partial correlations and trends across MBD/TDLU categories were calculated. Pregnenolone was positively associated with percent MBD-area (MBD-A, rho: 0.30; p-trend = 0.01) among premenopausal luteal phase women. Progesterone tended to be positively associated with percent MBD-A among luteal phase (rho: 0.26; p-trend = 0.07) and postmenopausal (rho: 0.17; p-trend = 0.04) women. Consistent with experimental data, implicating an elevated 5α-pregnanes/3α-dihydroprogesterone (5αP/3αHP) metabolite ratio in breast cancer, higher 5αP/3αHP was associated with elevated percent MBD-A among luteal phase (rho: 0.29; p-trend = 0.08), but not postmenopausal women. This exploratory analysis provided some evidence that endogenous progesterone and progesterone metabolites might be correlated with MBD, a strong breast cancer risk factor, in both pre- and postmenopausal women undergoing breast biopsy. Additional studies are needed to understand the role of progesterone/progesterone metabolites in breast tissue composition and breast cancer risk.

## 1. Introduction

**Figure 1:**
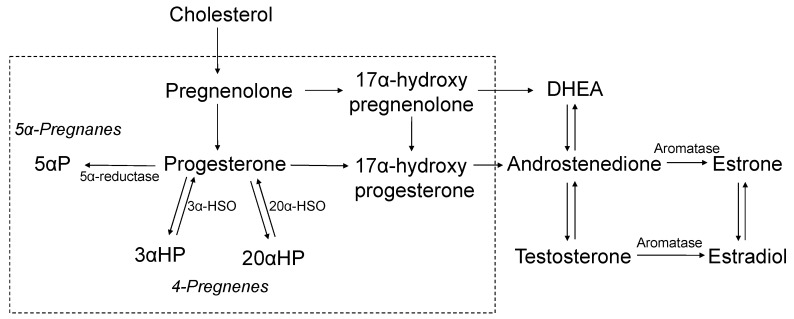
Schematic of the synthesis of sex steroid hormones from cholesterol. In normal breast tissue, pregnenes (progesterone is a pregnene) are the predominant compounds. All of the 4-pregnenes (not shown) can be irreversibly converted to 5α-pregnane (respectively) via 5α-reductase. Experimental studies in mice have shown that the two metabolites, 5αP and 3αHP, show the greatest differences between tumor and non-tumor samples; the ratio of 5αP/3αHP is more than 10-fold higher in breast tumor tissues and 3-times higher in circulation, comparing mice that developed tumors to mice without tumors [18]. Laboratory data has shown that activities of the progesterone metabolites are similar by age and by ER-status [9]. The current assay measured the 7 progesterone-related compounds enclosed in the box as follows: pregnenolone, progesterone, 17α-hydroxypregnenolone, 17α-hydroxyprogesterone, and select progesterone metabolites (5αP, 3αHP, and 20αHP). Estradiol was measured previously, using an independent assay [19]. Abbreviations: 5αP, 5α-dihydroprogesterone; 3αHP, 3α-dihydroprogesterone; 20αHP, 20α-dihydroprogesterone; 3α-HSO, 3α-hydroxysteroid oxidoreductase; 20α-HSO, 20α-hydroxysteroid oxidoreductase; DHEA, dehydroepiandrosterone.

Although extensive epidemiologic and experimental evidence implicate endogenous estrogen in breast cancer risk [1,2,3,4,5,6], progesterone is also thought to play a significant role in breast cancer development [7,8]. Laboratory data suggest that an imbalance in circulating sex steroid hormones, such as estrogen and progesterone levels, is crucial for breast carcinogenesis. Progesterone is synthesized from its precursor pregnenolone (Figure 1) and subsequently metabolized into 1) 4-pregnenes: metabolites that retain their double bond and 2) 5α-pregnanes: metabolites in which 5α-reductase has reduced the double bond [9]. The relative distribution of 4-pregnenes and 5α-pregnanes has been shown to be different in normal and malignant breast tissues whereby the 4-pregnanes [3α-dihydroprogesterone (3αHP) and 20α-dihydroprogesterone (20αHP)] predominate in normal tissue, while the 5α-pregnanes (5αP) are more abundant in tumor tissue [9,10,11]. Importantly, a higher ratio of 5αP to 3αHP is hypothesized to promote tumorigenesis. While experimental data indicate an important role for progesterone and its metabolites in breast cancer, there is limited epidemiologic data relating these hormones to breast cancer risk or its risk factors (i.e., mammographic breast density (MBD) or terminal ductal lobular unit (TDLU) involution) [12,13,14,15,16,17,18].

MBD and TDLU involution are radiologic and histologic measures of breast tissue composition, respectively, that have been demonstrated to be independent risk factors for the development of breast cancer [20]. Both MBD and TDLU involution are thought to reflect cumulative exposure to breast cancer risk factors, including endogenous hormones; as such, their study may have implications for understanding mechanisms underlying breast carcinogenesis. For example, increased MBD and delayed TDLU involution have been associated with hormonally-related breast cancer risk factors, such as nulliparity, later age at first birth, and later age at menopause [21,22]. However, studies evaluating the relation of circulating progesterone with MBD have largely yielded inconsistent findings [13,16,23,24,25,26,27,28,29], with the majority reporting null associations [23,26,27,29], and no prior studies have evaluated progesterone metabolites in relation to MBD or TDLU involution. Khodr et al. previously reported that higher levels of circulating progesterone (as measured with a chemiluminescent immunometric assay) were associated with increased TDLU involution (lower TDLU counts) in premenopausal women; similar patterns of association were observed among postmenopausal women, but results were not statistically significant [12]. The comprehensive study of progesterone and its metabolites among pre- and postmenopausal women has only recently become technically feasible with highly reproducible liquid chromatography-tandem mass spectrometry (LC-MS/MS) methods [30]. Leveraging this recently developed assay [30], we aimed to explore cross-sectional relationships between serum progesterone/progesterone metabolites, with a particular emphasis on the metabolites 5αP, 3αHP, and the ratio of 5αP-to-3αHP, with MBD and TDLU involution among women, attending a mammographic screening program, who were subsequently referred for image-guided diagnostic breast biopsy. As the biological effects of progesterone may be dependent upon dose and duration of exposure to other hormones, like estrogen [7], we also utilized pre-existing data on serum unconjugated estradiol levels [19] among these women and evaluated the progesterone-to-estradiol ratio.

## 2. Methods

### 2.1. Study Population

The National Cancer Institute (NCI) Breast Radiology Evaluation and Study of Tissues (BREAST) Stamp Project is a cross-sectional molecular epidemiologic study of MD conducted among 465 women, aged 40 to 65 years, who attended a U.S. mammographic screening program and were subsequently referred for diagnostic image-guided breast biopsy based on an abnormal mammogram from 2007 through 2010 at the University of Vermont (UVM) College of Medicine and University of Vermont Medical Center (a U.S. Breast Cancer Surveillance Consortium Center), as described previously [31]. Participants had no prior history of breast cancer or receiving cancer treatment, had not undergone breast surgery within one year, did not have breast implants, and were not taking breast cancer chemoprevention. Participants completed a standard self-administered health history questionnaire.

Out of 465 women who consented to participate in the study, at least 1 vial of serum was collected from 346 (74%) women. Of these 346 women, 21 with missing single X-ray absorptiometry (SXA) mammographic density data, 29 who were current hormone users (menopausal hormone therapy/oral contraceptives), and 40 premenopausal women with missing information on menstrual cycle phase were excluded from the study. After exclusions, a total of 256 women (premenopausal luteal phase: *n* = 65; follicular phase: *n* = 88; and postmenopausal: *n* = 103) were included in the final analytic population.

A woman was categorized as postmenopausal if she reported that her menstrual cycle stopped more than 12 months prior to the interview, she had undergone bilateral oophorectomy, or she had undergone hysterectomy, and she was 55 years of age or older; otherwise, a woman was categorized as premenopausal. Menstrual cycle length was determined by computing the difference in days between the self-reported date of the last menstrual period at the time of blood collection and the first day of the next menstrual period, which was reported via a postcard returned after blood collection, i.e., backward dating. For the binary classification of the menstrual cycle phase, women were categorized as luteal if the blood was collected <13 days prior to the start of the next menstrual cycle, and those remaining were categorized as follicular.

The postcard-ascertained day of the next menstrual period is a strong way to indicate the phase of the participants’ cycles. However, several women did not return their postcards. As such, blood samples (*n* = 28) with missing information on the next menstrual period were assigned to the binary follicular/luteal classification as follows: (1) *n* = 11 women who donated blood on days 1–5 of their current menstrual cycle (based on date of last menstrual period) were assigned to follicular, or (2) *n* = 17 were classified based on the concentration of measured hormone levels: *n* = 12 were classified as follicular phase since their unconjugated estradiol level was greater than or equivalent to their progesterone level, and *n* = 5 were classified as luteal since their progesterone level was substantially higher than unconjugated estradiol (mean progesterone was higher than unconjugated estradiol by approximately 2000 pmol/L).

### 2.2. Mammographic Breast Density Assessment

Digital raw mammographic images were transferred to the University of California at San Francisco for the quantitative area and volumetric MBD assessment, as previously described [31]. This analysis was restricted to prebiopsy craniocaudal views of the ipsilateral breast from the mammograms taken closest in time prior to breast biopsy date. Area MBD measures (MBD-A) were estimated by computer-assisted thresholding software [32,33]. Absolute MBD-A (cm^2^) was measured by setting a pixel threshold for dense tissue. Percent MBD-A was calculated by dividing absolute MBD-A by total breast area and multiplying by 100. To estimate MBD as a fibroglandular volume (MBD-V), an SXA breast density phantom was affixed to the mammographic compression paddle and included in the X-ray field, and the calibrated grayscale pixel values in the breast image were used to estimate absolute (cm^3^) and percent MBD-V measures [34]. SXA test phantoms demonstrated a repeatability standard deviation of 2%, with a ± 2% accuracy for the entire thickness and MBD ranges [34].

### 2.3. Histologic Assessment of TDLU Involution

TDLU involution was quantified in background normal breast biopsy tissue using reliable, standardized measures [21,35]. A study pathologist enumerated normal TDLUs on H&E-stained tissue sections that were digitized at X20 magnification (Aperio ScanScope CS) and were prepared for web-based viewing, as previously described [35]; to derive TDLU counts/100 mm^2^, the lasso tool in Digital Image Hub (SlidePath/Leica, Dublin, Ireland) was used to manually outline and measure total tissue area (mm^2^). For women with observed TDLUs, up to 10 were evaluated, and the maximum diameter (TDLU span) was measured with an electronic ruler in microns [36]. TDLU analyzer software [12,37] measured the number of acini, secretory substructures within TDLUs, and median acini counts/TDLU were selected as a summary measure for each woman. Higher TDLU counts, larger TDLU span, and higher acini counts/TDLU are indicative of delayed TDLU involution [38], a strong breast cancer risk factor [38,39,40].

### 2.4. Blood Collection and Laboratory Assay

Whole blood samples were collected using standard techniques, allowed to clot for 30 min, and processed at the UVM General Clinical Research Center. Samples were centrifuged at 3000 rpm for 15 min, and serum was aliquoted into 2 mL cryovials and frozen at −80 °C until shipment to the NCI biorepository, where vials were stored in liquid nitrogen. Endogenous pregnenolone, progesterone, and their major metabolites were measured using a stable isotope dilution high-performance LC-MS/MS assay at the Protein Characterization Laboratory, Frederick National Laboratory for Cancer Research [30]. This assay simultaneously measured pregnenolone, 17α-hydroxypregnenolone, progesterone, 17α-hydroxyprogesterone, 5αP, 3αHP, and 20αHP. Within batch and between batch, coefficients of variation (CV) for all the metabolites were <5%, and intraclass correlations (ICC) for all hormones measured were above 92% based on blinded duplicates. We utilized pre-existing data on unconjugated estradiol, previously measured using LC-MS/MS [19], from the same pre-biopsy blood draw used for assessment of progesterone metabolites to evaluate the progesterone-to-estradiol ratio.

### 2.5. Statistical Analysis

All analyses were stratified by menopausal status (premenopausal or postmenopausal) and menstrual cycle phase for premenopausal women (luteal or follicular). We evaluated associations of individual progesterone and progesterone-related metabolites with MBD and TDLU metrics. We also evaluated the ratios of 5αP-to-3αHP and progesterone-to-unconjugated estradiol with these metrics. The hormones were modeled as natural logarithm transformed continuous variable, and the MBD and TDLU measures were categorized into tertiles (T1, T2, and T3). Analysis of covariance was used to examine age and body mass index (BMI)-adjusted geometric means (GM) and 95% confidence intervals (CI) of progesterone and progesterone-related metabolites levels (pmol/L) across tertile categories of MBD and TDLU metrics. Age and BMI-adjusted Spearman’s partial rank correlations between progesterone/progesterone-related metabolites and MBD/TDLU measures were also calculated. We further adjusted analyses among luteal phase women for day in the cycle to account for variation in progesterone levels across the luteal phase as progesterone production increases. We evaluated potential confounding by assessing the relationship between reproductive and other risk factors previously known to be associated with MBD, TDLU involution, and progesterone and progesterone metabolites. Smoking and race were identified as potential confounders, but their adjustment did not substantively change results; therefore, we reported results with age and BMI adjustment. In a sensitivity analysis, we repeated analyses after excluding women who were diagnosed with breast carcinoma (in situ or invasive) at biopsy (luteal: *n* = 7; follicular: *n* = 12; and postmenopausal: *n* = 25). We also performed a sensitivity analysis among premenopausal women who reported regular menstrual cycle lengths (24–34 days, luteal: *n* = 47; follicular: *n* = 59). All statistical tests were two-sided with a 5% type I error; given the exploratory nature of this analysis, we also discussed consistent patterns of association across correlated outcomes (e.g., MBD metrics). Analyses were conducted with SAS software version 9.4 (SAS Institute Inc., Cary, NC, USA).

## 3. Results

The mean ages at biopsy were 45.9 (standard deviation (SD): 3.8), 46.9 (3.8), and 57.3 (4.3) for premenopausal women in the luteal phase, follicular phase, and postmenopausal women, respectively (Table 1). Overall, the vast majority of women had benign diagnoses (*n* = 212, 82.8%), with the distribution of diagnoses as follows: benign non-proliferative (*n* = 89, 34.8%), benign proliferative without atypia (*n* = 106, 41.4%), benign proliferative with atypia (*n* = 17, 6.6%), in-situ/invasive lesions (*n* = 44, 17.2%). Study participants were predominantly white (luteal: 96.9%; follicular: 93.2%; postmenopausal: 92.2%). Compared to premenopausal women, postmenopausal women were more likely to be obese and to have ever smoked cigarettes.

As expected, the circulating levels of progesterone-related metabolites were higher in both menstrual cycle phases of premenopausal women compared to postmenopausal women (Table 2). Among premenopausal women, circulating levels of progesterone-related metabolites were lower in the follicular phase and higher in the luteal phase. Among luteal phase women, progesterone levels were observed in the highest concentration (14,405 pmol/L); whereas, among follicular phase and postmenopausal women, 17α-hydroxypregnenolone levels were observed in the highest concentrations (3414 pmol/L and 2625 pmol/L, respectively). The concentration of circulating levels of 3αHP was the lowest across all groups of women. As expected, MBD and TDLU measures were higher in premenopausal women compared to postmenopausal women and did not vary by menstrual cycle phase.

Among premenopausal women, strong, positive correlations between progesterone and its metabolite 20αHP were observed (luteal: correlation (rho) = 0.89; follicular: rho = 0.76; correlation *p*-values < 0.0001) (Supplementary Table S1). Progesterone and 5αP were highly correlated (rho = 0.72; *p*-value < 0.0001) in luteal phase samples, but not in follicular phase samples (rho = 0.02, *p*-value = 0.08). Among postmenopausal women, moderate positive correlations were observed between progesterone and the following hormones: pregnenolone (rho = 0.62, *p*-value < 0.0001), 17α-hydroxyprogesterone (rho = 0.69, *p*-value ≤ 0.0001), and 20αHP (rho = 0.64, *p*-value < 0.0001).

### 3.1. Luteal Menstrual Cycle Phase Women (n = 65)

**Figure 2:**
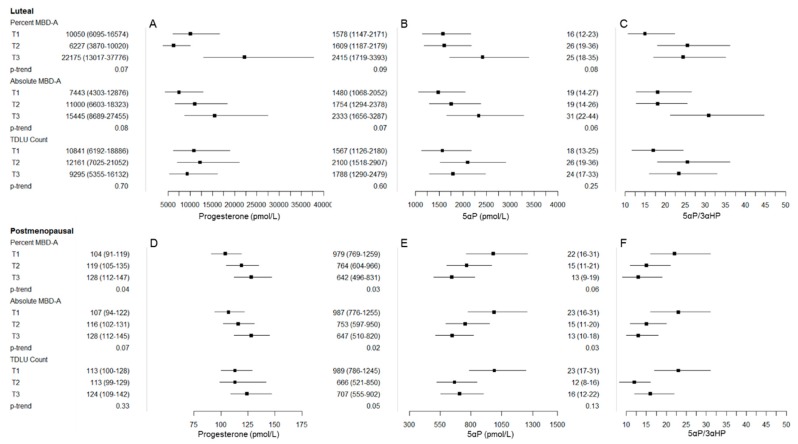
Graphical representation of major findings/patterns of association between progesterone, 5α-pregnanes (5αP), and 5αP/3α-dihydroprogesterone (3αHP) with mammographic breast density (MBD) and terminal duct lobular unit (TDLU) measures among luteal phase premenopausal women (panels **A**: progesterone, **B**: 5αP, and **C**: 5αP/3αHP) and postmenopausal women (panels **D**: progesterone, **E**: 5αP, and **F**: 5αP/3αHP). The box represents the geometric mean (GM), and whiskers represent the 95% confidence interval (CI) across tertile of percent MBD-A, absolute MBD-A, and TDLU count (from top to bottom in each panel). In panels **A** and **D**, progesterone tended to be positively associated with MBD measures in both the luteal phase (panel **A**) and postmenopausal (panel **D**) women. In contrast, patterns for 5αP and 5αP/3αHP were opposite in luteal phase (panels **B** and **C**) and postmenopausal (panels E and F) women, with positive patterns of association among luteal phase women and inverse patterns of association in postmenopausal women.

Pregnenolone levels increased across increasing tertiles of percent MBD-A (T3 vs. T1: 5095 vs. 3438, p-trend = 0.01; rho = 0.30, *p*-value = 0.02) (Table 3). There was a suggestion of a positive association between progesterone and the majority of MBD measures (Table 3), with patterns of association for percent and absolute measures of MBD-A, shown in Figure 2: percent MBD-V (T3 vs. T1: 14036 vs. 8260, p-trend = 0.28; rho = 0.15, *p*-value = 0.25); percent MBD-A (T3 vs. T1: 22175 vs. 10050, p-trend = 0.07; rho = 0.26, *p*-value = 0.06); absolute MBD-V (T3 vs. T1: 15451 vs. 7791, p-trend = 0.11; rho = 0.14, *p*-value = 0.29); absolute MBD-A (T3 vs. T1: 15445 vs. 7443, p-trend = 0.08; rho = 0.20, *p*-value = 0.13). 5αP also tended to be positively associated with all MBD measures (Table 3, Figure 2): percent MBD-V (T3 vs. T1: 2169 vs. 1682, p-trend = 0.39; rho = 0.17, *p*-value = 0.21); percent MBD-A (T3 vs. T1: 2415 vs. 1578, p-trend = 0.09; rho = 0.24, *p*-value = 0.08); absolute MBD-V (T3 vs. T1: 1978 vs. 1504, p-trend = 0.25; rho = 0.13, *p*-value = 0.34); absolute MBD-A (T3 vs. T1: 2333 vs. 1480, p-trend = 0.07; rho = 0.23, *p*-value = 0.09). 3αHP did not have a clear pattern of association with MBD (Table 3). 

5αP/3αHP tended to be positively associated with percent MBD-V (T3 vs. T1: 24 vs. 19, p-trend = 0.40; rho = 0.20, *p*-value = 0.15), percent MBD-A (T3 vs. T1: 25 vs. 16, p-trend = 0.08; rho = 0.29, *p*-value = 0.03), and absolute MBD-A (T3 vs. T1: 31 vs. 19, p-trend = 0.06; rho = 0.21, *p*-value = 0.12) (Table 3, Figure 2). In addition, the ratio of progesterone/unconjugated estradiol was suggestive of a positive association with absolute MBD-V (T3 vs. T1: 101 vs. 35, p-trend = 0.05; rho = 0.21, *p*-value = 0.16). No clear patterns of association were observed between 17α-hydroxypregnenolone, 17α-hydroxyprogesterone, and 20αHP and MBD measures, or for progesterone/progesterone metabolites and TDLU measures among luteal phase women.

### 3.2. Follicular Menstrual Cycle Phase Women (n = 88)

Patterns of association between progesterone-related metabolites and MBD in follicular phase women were not as consistent as patterns in luteal phase women (Table 4). 5αP tended to be inversely associated with percent MBD-A (T3 vs. T1: 579 vs. 977, p-trend = 0.02; rho = −0.24, *p*-value = 0.03) but not with percent MBD-V or absolute MBD measures. Similar to luteal phase women, 3αHP did not have a clear pattern of association with MBD. In contrast to associations in luteal phase women, 5αP/3αHP was not associated with percent MBD-A (T3 vs. T1: 14 vs. 21, p-trend = 0.11; rho = −0.16, *p*-value = 0.13). 17α-hydroxyprogesterone was positively associated (T3 vs. T1: 675 vs. 472, p-trend = 0.03; rho = −0.19, *p*-value = 0.09), and 20αHP was inversely associated with TDLU count/100 mm^2^ (T3 vs. T1: 180 vs. 275, p-trend = 0.02; rho = −0.22, *p*-value = 0.04). A suggestive positive association was observed between 3αHP and median acini count per TDLU (p-trend = 0.08; rho = 0.26, *p*-value = 0.06). The ratio of progesterone/unconjugated estradiol tended to be inversely associated with some MBD measures and all of the TDLU metrics.

### 3.3. Postmenopausal Women (n = 103)

We graphically plotted the major findings/patterns of association between progesterone, 5αP, and 5αP/3αHP for both luteal phase premenopausal and postmenopausal women to demonstrate the consistency of the suggested positive association for progesterone across both groups and the opposite pattern of association for 5αP and 5αP/3αHP (Figure 2). In particular, progesterone tended to be positively associated with all MBD measures in postmenopausal women: percent MBD-V (T3 vs. T1: 120 vs. 107, p-trend = 0.28; rho = 0.17, *p*-value = 0.10); percent MBD-A (T3 vs. T1: 128 vs. 104, p-trend = 0.04; rho = 0.17, *p*-value = 0.09); absolute MBD-V (T3 vs. T1: 122 vs. 105, p-trend = 0.12; rho = 0.19, *p*-value = 0.06); absolute MBD-A (T3 vs. T1: 128 vs. 107, p-trend = 0.07; rho = 0.15, *p*-value = 0.13) (Table 5). 5αP was inversely associated with percent MBD-A (T3 vs. T1: 642 vs. 979, p-trend = 0.03; rho = −0.23, *p*-value = 0.02) and absolute MBD-A (T3 vs. T1: 647 vs. 987, p-trend = 0.02; rho = −0.25, *p*-value = 0.01). 5αP/3αHP was inversely associated with percent MBD-A (T3 vs. T1: 13 vs. 22, p-trend = 0.06; rho = −0.21, *p*-value = 0.04) and absolute MBD-A (T3 vs. T1: 13 vs. 23, p-trend = 0.03; rho = −0.23, *p*-value = 0.02). Similar to associations with MBD measures, suggestive inverse associations between 5αP and 5αP/3αHP with TDLU count/100 mm^2^ and median TDLU span were observed. We did not observe a clear pattern of association between pregnenolone, 17α-hydroxypregnenolone, 17α-hydroxyprogesterone, and 20αHP and MBD/TDLU measures in postmenopausal women. The progesterone/unconjugated estradiol ratio was positively associated with percent MBD-A (T3 vs. T1: 16 vs. 9, p-trend = 0.03; rho = 0.24, *p*-value = 0.02).

### 3.4. Sensitivity Analyses

Results were similar after excluding women diagnosed with in situ or invasive carcinoma at biopsy among both premenopausal and postmenopausal women (Supplementary Tables S2–S5). Upon restricting analyses to premenopausal women who reported regular menstrual cycle lengths, the results were consistent with those derived from the full analysis of luteal and follicular phase women (Supplementary Tables S6 and S7, respectively).

## 4. Discussion

In this cross-sectional study of women undergoing diagnostic breast biopsy after routine mammographic screening, we assessed the relationship between circulating progesterone/progesterone metabolites with breast cancer risk factors: MBD and TDLU involution. There was a suggestive positive association between progesterone with MBD measures, particularly when measured during the luteal phase in premenopausal women and among postmenopausal women. Consistent with experimental data, implicating an elevated 5αP/3αHP ratio in breast cancer, we found that higher 5αP/3αHP tended to be associated with elevated MBD among luteal phase premenopausal, but not postmenopausal, women. Among postmenopausal women, 5αP and the 5αP/3αHP ratio were inversely associated with both MBD and TDLU measures. We also observed a novel positive association between pregnenolone and percent MBD-A among luteal phase women, which requires future study. These findings suggest the need for additional studies in both pre- and postmenopausal women to better understand the role of progesterone and its metabolites in breast tissue composition and breast cancer risk.

Several lines of evidence suggest that early menarche, shorter menstrual cycle length, and late menopause increase the cumulative exposure of the mammary gland to progesterone, thereby increasing the risk of breast cancer [7]. The proliferation of breast epithelium during the luteal phase has been attributed to the mitogenic activity of progesterone [41,42,43]. Thus, increased time spent in the luteal phase due to a higher number of lifetime menstrual cycles (i.e., earlier onset of menarche and delayed menopause) may increase the risk of breast cancer. In support of this notion, the current study demonstrated a positive association between circulating progesterone and MBD measures among luteal phase women. Two prior studies also found positive associations between progesterone and MBD among luteal phase women [13,24], whereas two studies reported null associations [23,26], and one recent study observed an inverse association between progesterone and MBD [25]. The inconsistencies across studies may be due to variations in the study populations, including age, assays used, and/or the MBD assessment methods. Our study population included women aged 40–65 years, and thus many who we considered premenopausal might well be perimenopausal and experiencing unpredictable fluctuations in estrogens and progesterone. Nonetheless, results from sensitivity analyses restricted to those premenopausal women who reported regular menstrual cycle lengths were largely consistent with those observed overall.

Similar to our findings for luteal phase premenopausal women, we also observed a consistent pattern of positive associations between progesterone and MBD measures among postmenopausal women. Our findings for postmenopausal women were consistent with one prior study, which also identified a positive association between progesterone and MBD [28], but were in contrast with three prior studies, which reported null associations [23,27,29]. Notably, all four prior studies used radioimmunoassays to measure progesterone, assays that are not sensitive to the low progesterone levels seen in this age group. For example, in prior studies of postmenopausal women using chemiluminescent and radioimmunoassays, nondetectable levels of progesterone ranged from 30% of samples in one study [27] to 80% of samples in another [12]. In our study, all progesterone/progesterone metabolites were above the limit of detection (0.1 ng/dl) of the LC-MS/MS assay.

MBD reflects the relative proportions of stroma, epithelium, and adipose tissues in the breast, with histologic studies demonstrating that elevated MBD is predominantly stroma [44,45]. Progesterone stimulates the proliferation of stromal tissue [46,47] and is a major regulator of cell proliferation and stem cell activation in adult mammary gland development [43]. Additionally, binding of progesterone to the progesterone receptor (PR) activates downstream NFκ-B and Wnt4 signaling pathways that contribute to breast tumorigenesis [7,43]. Thus, the positive association between progesterone and MBD, seen in our study, might be due to progesterone’s proliferative effects on the stroma or alternatively through activation of the PR signaling pathways.

Radiologic measures of MBD are also considered to reflect the underlying at-risk epithelium, represented by TDLUs [35,48]. TDLUs are anatomical epithelial structures from which breast cancer and its precursors originate [49]. As progesterone stimulates proliferation of acini [46], the secretory substructures within TDLUs, a positive association between progesterone and TDLU counts may be hypothesized; however, we observed largely null associations for both pre- and postmenopausal women. Our findings were in contrast to the inverse association between progesterone and TDLU counts among premenopausal women reported previously [12]. Differences in the age distribution of the two study populations may have contributed to the disparate findings; in the current study, premenopausal women were older than 45 years compared with 70% below age 40 in the prior study. In addition, the prior study was conducted in healthy donors undergoing research biopsies [12], whereas the current study was largely comprised of diagnostic breast biopsies from women with benign lesions. Due to the cross-sectional nature of our study design, we were unable to infer causal relationships of progesterone and progesterone metabolites with measures of breast tissue composition (i.e., TDLU involution and MBD) among this population of women referred for diagnostic breast biopsy. Thus, differences between studies in the window of exposure assessment of progesterone may account for the largely null trend we observed for the relation of progesterone with measures of TDLU involution in the setting of benign breast disease.

Progesterone is metabolized locally in both normal and malignant breast tissues [10], and among this population of women undergoing a diagnostic breast biopsy, our findings suggested for the first time that endogenous progesterone metabolites might be correlates of MBD. We observed an elevated 5αP/3αHP ratio associated with higher MBD among luteal phase premenopausal women, suggesting that the relationship between increased 5αP/3αHP and breast cancer in experimental models might be operating through elevated MBD. Our study further emphasized the importance of considering the menstrual cycle phase when measuring progesterone metabolites and suggested that higher progesterone, 5αP, and 5αP/3αHP might only be reflective of increased MBD, and possibly breast cancer risk, when measured in the luteal phase of premenopausal women.

In contrast to the experimental data and progesterone metabolite findings for luteal phase women in the current study, among postmenopausal women higher 5αP/3αHP was inversely associated with both MBD and TDLU measures. Interactions between progesterone metabolites, other circulating hormones, and the breast tissue microenvironment might contribute to the observed differences by menopausal status. Due to a large number of statistical comparisons, these findings might also be due to chance. Future larger studies assessing the role of progesterone metabolites in breast tissue composition, with serial measures spanning the menopausal transition, are needed.

The role of progesterone is tissue-specific, and it is known to act in concert with other hormones, particularly estrogen [50]. Both estrogen and progesterone are important for the normal development of the mammary gland [43]. An imbalance in these sex steroid hormones may contribute to the uncontrolled growth of breast tissue and thereby induce breast cancer. We also assessed the association of the ratio of progesterone to unconjugated estradiol with both MBD and TDLU involution and found a positive association with some MBD metrics among luteal phase women and postmenopausal women. Thus, the progesterone/unconjugated estradiol ratio may be an important predictor of MBD and warrants further investigation with respect to breast cancer risk.

We observed a novel association between pregnenolone and MBD-A among luteal phase women. Pregnenolone is a precursor of progesterone that can metabolize progesterone in breast tissue [51]. A recent study evaluating a steroid hormone profile in healthy breast adipose tissue around the tumor showed that pregnenolone was one of the predominant steroid hormones [52]. Pregnenolone in the surrounding tissue of the tumor may, therefore, act as a reservoir of progesterone that the tumor can access to facilitate proliferation. Additional work is needed to explore the role of pregnenolone in breast tissue composition and with breast cancer risk/risk factors.

Percent MBD is the proportion of dense fibroglandular tissue in the total breast area. Absolute MBD reflects the non-fatty fibroglandular tissue component. In general, we observed consistent relationships for progesterone/progesterone metabolites with percent and absolute MBD-A measures. Observed differences in the association between progesterone/progesterone metabolites and percent and absolute MBD (i.e., 5αP among follicular phase women) might be due in part to differences contributed by the breast tissue microenvironment.

A major strength of this study is that we used a highly sensitive and reproducible LC-MS/MS assay, particularly for postmenopausal women, as progesterone levels using other assay methods are often below the limit of detection. The LC-MS/MS assay measures progesterone metabolites—hypothesized to be important for mammary gland development—that are not measured by commercially available kits. Our study assessed both MBD and TDLU involution using quantitative reliable methods. MBD-V and MBD-A measures were previously shown to be highly correlated in this study population [31]. Data from the current study also supported this as both MBD-V and MBD-A associations with progesterone and progesterone-related hormones were largely consistent. This study focused on women undergoing digital mammography with clinically-indicated breast biopsies, who are therefore at increased risk of developing breast cancer; identifying biomarkers for risk prediction among these high-risk women is important. The menstrual cycle phase was determined by the postcard-ascertained day of the next menstrual period for the majority of premenopausal women (82%, 125/153); thus, potential misclassification in cycle phase due to missing menstrual cycle postcards was small. Our study also has several limitations. Women in our study were primarily white and highly educated, and our study population, consisting of women referred for clinically-indicated breast biopsy, might not generalize to a healthy population. Nevertheless, studying this high-risk population is important. In the US, about 1.6 million women undergo breast biopsy annually. The vast majority (~80%) of breast biopsies are found to be benign, with the remaining ~20% being in-situ/invasive lesions, and these proportions are consistent with the distribution of biopsy diagnoses observed in our study population. We measured progesterone metabolites in a single blood draw for each woman. Larger studies are needed to replicate our findings. It is possible that a change in progesterone/ progesterone metabolites across the menstrual cycle may be more reflective of patterns of MBD or TDLU measures; thus, additional research evaluating multiple hormone measures for an individual woman may be useful. A single measurement may not be representative of long-term measures. However, a separate reproducibility study of the hormones measured in this study observed moderate to high stability in samples collected two years apart; of note, the ICC (95% CI) for progesterone was 0.84 (0.65–0.93) among postmenopausal women (Geczik et al., submitted). It is also plausible that differences in the direction of associations between progesterone/progesterone metabolites and MBD/TDLU metrics across menstrual cycle phases and menopausal status might be a due chance. As this was the first study to investigate relationships between serum progesterone/progesterone metabolites, MBD, and TDLU involution and was of exploratory nature, results were not adjusted for multiple comparisons. Our findings would not be considered statistically significant after formal multiple testing adjustment, likely due to limited sample size or potential modest effects of progesterone and progesterone-related metabolites on MBD and TDLU measures.

In summary, our findings suggest that increased circulating progesterone is associated with elevated MBD, particularly among luteal phase premenopausal women and postmenopausal women. Levels of endogenous progesterone metabolites were also associated with MBD, with higher 5αP/3αHP being associated with elevated MBD among luteal phase premenopausal, but not postmenopausal, women. Among this population of women undergoing a diagnostic breast biopsy, we observed little support for a role of progesterone/progesterone metabolites in TDLU involution. This exploratory analysis provided some evidence that endogenous progesterone and progesterone metabolites might be correlated with MBD in both pre- and postmenopausal women undergoing breast biopsy. Further research is warranted to explore interrelationships between progesterone metabolites and other circulating sex steroid hormones with breast tissue composition and breast cancer risk.

## Figures and Tables

**Table 1:** Selected characteristics of women in the Breast Radiology Evaluation and Study of Tissues (BREAST) Stamp Project stratified by menopausal status and menstrual cycle phase.

Characteristics	Luteal (*N* = 65)		Follicular (*N* = 88)		Postmenopausal (*N* = 103)	
	n ^1^	%	n ^1^	%	n ^1^	%
Age at biopsy among premenopausal women (years)						
<45	27	41.5	23	26.1	NA	NA
45–49	26	40.0	43	48.9	NA	NA
≥50	12	18.5	22	25.0	NA	NA
Age at biopsy among postmenopausal women (years)						
<55	NA	NA	NA	NA	30	29.1
55–59	NA	NA	NA	NA	38	36.9
≥60	NA	NA	NA	NA	35	34.0
Age at Menarche						
≤12	21	33.9	28	31.8	42	41.2
13	27	43.6	35	39.8	34	33.3
≥14	14	22.6	25	28.4	26	25.5
Race and ethnicity						
White, Non-Hispanic	63	96.9	82	93.2	95	92.2
Other	2	3.1	6	6.8	8	7.8
Body mass index (kg/m^2^)						
<25	36	55.4	43	48.9	41	39.8
25.0–29.9	15	23.1	21	23.9	31	30.1
≥30	14	21.5	24	27.3	31	30.1
Cigarette smoking						
Never	34	54.8	52	60.5	37	39.0
Former	24	38.7	26	30.2	47	50.0
Current	4	6.5	8	9.3	11	11.6
Oral contraceptive use						
Never	10	15.4	11	12.5	15	14.6
Former	55	84.6	77	87.5	88	85.4
Menopausal hormone use						
Never	59	92.2	80	90.9	67	65.1
Former	5	7.8	8	9.1	36	35.0
Age at menopause						
<45	NA	NA	NA	NA	17	18.7
45–49	NA	NA	NA	NA	26	28.6
≥50	NA	NA	NA	NA	48	52.8
Family history of breast cancer in a first-degree relative						
None	52	80.0	64	72.7	75	73.5
1 or more	13	20.0	24	27.3	27	26.5
Breast biopsy prior to enrollment						
Never	45	69.2	61	69.3	62	61.4
Ever	20	30.8	27	30.7	39	38.6
Biopsy diagnosis						
Benign non-proliferative	29	44.6	29	33.0	31	30.1
Benign proliferative with or without atypia ^2^	29	44.6	47	53.4	47	45.6
In-situ/Invasive	7	10.7	12	13.6	25	24.3

^1^ Columns may not sum to total because of missing values as follows: Luteal phase: Cigarette smoking: *n* = 3; Age at menarche: *n* = 3; Menopausal hormone use: *n* = 1; Follicular phase: Cigarette smoking: *n* = 2. Postmenopausal women: Cigarette smoking: *n*= 8; Age at menarche: *n* = 1; Menopausal hormone use: *n* = 1; Family history of breast cancer in a first-degree relative: *n* = 1. ^2^ Benign proliferative with atypia: Luteal *n* = 2; Follicular *n* =5; Postmenopausal *n* = 10.

**Table 2:** Distribution of progesterone, progesterone metabolites, unconjugated estradiol, MBD measures, and TDLU measures of women in the BREAST Stamp Project, stratified by menopausal status and menstrual cycle phase.

	Luteal (*N* = 65)		Follicular (*N* = 88)		Postmenopausal (*N* = 103)	
	Median	(IDR)	Median	(IDR)	Median	(IDR)
Hormones (pmol/L)						
Pregnenolone	3955	2334–6988	2474	1313–5572	1796	908–3151
17α-hydroxypregnenolone	3632	2353–8492	3414	1858–10064	2625	1569–6078
Progesterone	14405	2058–40437	278	123–1547	110	77–181
17α-hydroxyprogesterone	2169	796–3775	565	285–1658	361	195–819
3α-dihydroprogesterone (3αHP)	83.3	30–209	42.5	23–93	45.5	25–102
5α-dihydroprogesterone (5αP)	1990	769–5172	807	300–1985	797	287–1914
20α-Dihydroprogesterone (20αHP)	4211	545–10612	216	116–581	111	65–186
5αP/3αHP Ratio	23.2	8.5–55	18.2	4.85–57	15.9	4.3–70.6
Unconjugated estradiol (E2)	237	98–717	220	23–605	8.1	2.5–42.9
Progesterone/E2 ratio	84.8	4.1–190	1.8	0.40–17	13.5	2.4–40
	**Mean**	**SD**	**Mean**	**SD**	**Mean**	**SD**
MBD measures						
Percent MBD-V	43.3	20.2	45.3	23.0	30.3	17.2
Percent MBD-A	30.9	18.9	34.6	21.5	20.1	16.7
Absolute MBD-V	208.7	110.6	213.3	108.9	184	87.9
Absolute MBD-A	37.5	25.7	42.3	28.7	27.9	21.8
TDLU involution measures						
TDLU count/100 mm^2^	28.7	39.0	21.6	29.6	13.3	23.2
Median TDLU span, μ	317.4	112.1	304.0	93.9	227	90.1
Median acini count per TDLU	16.2	9.5	17.2	11.2	9.5	5.0

Abbreviations: IDR, interdecile range; MBD, mammographic breast density; TDLU, terminal ductal lobular unit; SD, standard deviation; MBD-V, mammographic breast density-volume; MBD-A, mammographic breast density-area.

**Table 3:** Relationships of MBD and TDLU measures with circulating geometric mean concentrations of progesterone and progesterone metabolites (pmol/L) in luteal phase women.

		Pregnenolone	17a-Hydroxypregnenolone	Progesterone	17α-Hydroxyprogesterone	3αHP	5αP	20αHP	5αP/3αHP	E2	Progesterone/E2
	*N*	GM ^1^ (LCI-UCI)	GM ^1^ (LCI-UCI)	GM ^1^ (LCI-UCI)	GM ^1^ (LCI-UCI)	GM ^1^ (LCI-UCI)	GM ^1^ (LCI-UCI)	GM ^1^ (LCI-UCI)	GM ^1^ (LCI-UCI)	GM ^1^ (LCI-UCI)	GM ^1^ (LCI-UCI)
**MBD measures**											
**Percent MBD-V**											
T1	29	3710 (2987–4607)	4069 (3177–5211)	8260 (4464–15,284)	1714 (1307–2248)	89 (63–126)	1682 (1165–2428)	2729 (1612–4620)	19 (13–28)	229 (152–343)	41 (18–92)
T2	30	3726 (3092–4492)	3999 (3231–4950)	10,688 (6286–18,172)	1845 (1460–2332)	69 (51–93)	1637 (1192–2247)	3453 (2193–5436)	24 (17–33)	202 (143–284)	49 (25–96)
T3	29	4548 (3647–5671)	3771 (2931–4852)	14,036 (7496–26,280)	2024 (1535–2669)	89 (62–126)	2169 (1492–3154)	4017 (2349–6868)	24 (17–36)	280 (197–398)	55 (27–113)
P-trend ^2^		0.24	0.70	0.28	0.44	0.99	0.39	0.36	0.40	0.44	0.61
Corr ^1^ (rho, *p* ^2^)		(0.12, 0.36)	(−0.09, 0.51)	(0.15, 0.25)	(0.08, 0.53)	(0.06, 0.67)	(0.17, 0.21)	(0.16, 0.23)	(0.20, 0.15)	(0.06, 0.67)	(0.07, 0.65)
**Percent MBD-A**											
T1	29	3438 (2868–4120)	3844 (3086–4789)	10,050 (6095–16,574)	1816 (1446–2281)	96 (72–129)	1578 (1147–2171)	3231 (2125–4913)	16 (12–23)	203 (143–288)	50 (25–98)
T2	30	3672 (3091–4362)	3873 (3143–4773)	6227 (3870–10,020)	1532 (1234–1903)	61 (46–81)	1609 (1187–2179)	2015 (1353–3001)	26 (19–36)	217 (158–297)	30 (16–56)
T3	29	5095 (4201–6178)	4164 (3295–5262)	22,175 (13,017–37,776)	2394 (1878–3052)	96 (70–132)	2415 (1719–3393)	6485 (4151–10,131)	25 (18–35)	295 (214–406)	75 (40–140)
P-trend ^2^		0.01	0.64	0.07	0.15	0.91	0.09	0.06	0.08	0.13	0.36
Corr ^1^ (rho, *p* ^2^)		(0.30, 0.02)	(0.07, 0.59)	(0.26, 0.06)	(0.21, 0.12)	(0.04, 0.79)	(0.24, 0.08)	(0.27, 0.04)	(0.29, 0.03)	(0.16, 0.29)	(0.15, 0.33)
**Absolute MBD-V**											
T1	29	3758 (3096–4561)	4618 (3737–5708)	7791 (4559–13,314)	1700 (1339–2159)	76 (56–103)	1504 (1090–2077)	2535 (1612–3987)	20 (14–28)	222 (163–303)	35 (20–63)
T2	30	4124 (3384–5025)	3513 (2830–4360)	10,507 (6083–18,149)	1894 (1484–2416)	77 (56–105)	2006 (1444–2787)	3068 (1933–4870)	26 (19–37)	265 (191–367)	41 (22–75)
T3	29	4031 (3252–4997)	3755 (2969–4749)	15,451 (8531–27,986)	1995 (1531–2600)	94 (67–131)	1978 (1383–2828)	4996 (3024–8253)	21 (15–31)	225 (150–335)	101 (47–214)
P-trend ^2^		0.61	0.17	0.11	0.38	0.42	0.25	0.07	0.70	0.85	0.05
Corr ^1^ (rho, *p* ^2^)		(−0.03, 0.81)	(−0.22, 0.11)	(0.14, 0.29)	(0.15, 0.28)	(0.15, 0.26)	(0.13, 0.34)	(0.18, 0.17)	(−0.04, 0.78)	(0.04, 0.81)	(0.21, 0.16)
**Absolute MBD-A**											
T1	29	3332 (2751–4037)	4020 (3232–5001)	7443 (4303–12,876)	1675 (1312–2139)	77 (56–105)	1480 (1068–2052)	2421 (1520–3858)	19 (14–27)	189 (137–260)	45 (24–85)
T2	30	4403 (3683–5264)	4427 (3612–5424)	11,000 (6603–18,323)	2019 (1608–2536)	92 (68–123)	1754 (1294–2378)	3413 (2212–5267)	19 (14–26)	270 (195–374)	35 (19–67)
T3	29	4225 (3454–5166)	3365 (2676–4231)	15,445 (8689–27,455)	1869 (1446–2417)	75 (54–104)	2333 (1656–3287)	4692 (2877–7649)	31 (22–44)	267 (190–375)	73 (37–144)
P-trend ^2^		0.10	0.30	0.08	0.54	0.93	0.07	0.06	0.06	0.15	0.33
Corr ^1^ (rho, *p* ^2^)		(0.13, 0.34)	(−0.13, 0.35)	(0.20, 0.13)	(0.09, 0.49)	(0.03, 0.82)	(0.23, 0.09)	(0.23, 0.09)	(0.21, 0.12)	(0.14, 0.35)	(0.16, 0.29)
**TDLU involution measures**											
**TDLU count/100 mm^2^**											
T1	28	3543 (2915–4307)	3853 (3084–4813)	10,814 (6192–18,886)	1828 (1430–2335)	88 (64–120)	1567 (1126–2180)	3533 (2210–5649)	18 (13–25)	194 (138–273)	51 (25–104)
T2	29	4094 (3379–4961)	3865 (3105–4811)	12,161 (7025–21,052)	1949 (1532–2481)	81 (60–111)	2100 (1518–2907)	4092 (2579–6495)	26 (19–36)	315 (234–423)	38 (20–70)
T3	29	4291 (3538–5204)	4133 (3317–5150)	9295 (5355–16,132)	1788 (1404–2279)	75 (55–103)	1788 (1290–2479)	2598 (1633–4132)	24 (17–33)	206 (150–282)	60 (31–116)
P-trend ^2^		0.18	0.66	0.70	0.90	0.50	0.60	0.36	0.25	0.90	0.71
Corr ^1^ (rho, *p* ^2^)		(0.12, 0.39)	(0.08, 0.56)	(−0.07, 0.60)	(−0.07, 0.62)	(−0.04, 0.76)	(0.03, 0.82)	(−0.11, 0.40)	(0.11, 0.43)	(−0.01, 0.95)	(0.04, 0.82)
**Median TDLU span, μ**											
T1	19	4606 (3756–5649)	4350 (3369–5616)	12,282 (7052–21,393)	2009 (1592–2537)	79 (58–108)	1796 (1244–2595)	3858 (2370–6283)	23 (16–33)	328 (224–481)	37 (18–74)
T2	20	3691 (3029–4498)	3554 (2775–4553)	8942 (5224–15,306)	1735 (1384–2174)	77 (57–104)	2103 (1473–3004)	2630 (1640–4217)	27 (19–39)	218 (151–315)	53 (27–103)
T3	20	4358 (3551–5347)	4109 (3180–5309)	11,030 (6323–19,239)	1754 (1389–2216)	83 (61–114)	1674 (1158–2420)	3267 (2004–5326)	20 (14–29)	210 (141–312)	54 (26–112)
P-trend ^2^		0.71	0.76	0.79	0.42	0.81	0.79	0.64	0.64	0.11	0.45
Corr ^1^ (rho, *p* ^2^)		(−0.05, 0.77)	(−0.05, 0.76)	(−0.03, 0.85)	(−0.12, 0.44)	(0.04, 0.81)	(−0.06, 0.70)	(−0.10, 0.51)	(−0.15, 0.34)	(−0.22, 0.19)	(0.11, 0.52)
**Median acini count per TDLU**											
T1	18	4606 (3756–5649)	4350 (3369–5616)	12,282 (7052–21,393)	2009 (1592–2537)	79 (58–108)	1796 (1244–2595)	3858 (2370–6283)	23 (16–33)	328 (224–481)	37 (18–74)
T2	19	3691 (3029–4498)	3554 (2775–4553)	8942 (5224–15,306)	1735 (1384–2174)	77 (57–104)	2103 (1473–3004)	2630 (1640–4217)	27 (19–39)	218 (151–315)	53 (27–103)
T3	18	4358 (3551–5347)	4109 (3180–5309)	11,030 (6323–19,239)	1754 (1389–2216)	83 (61–114)	1674 (1158–2420)	3267 (2004–5326)	20 (14–29)	210 (141–312)	54 (26–112)
P-trend ^2^		0.71	0.76	0.79	0.42	0.81	0.79	0.64	0.64	0.11	0.45
Corr ^1^ (rho, *p* ^2^)		(−0.05, 0.77)	(−0.05, 0.76)	(−0.03, 0.85)	(−0.12, 0.44)	(0.04, 0.81)	(−0.06, 0.70)	(−0.10, 0.51)	(−0.15, 0.34)	(−0.22, 0.19)	(0.11, 0.52)

^1^ Correlation adjusted for age, body mass index, and day in the menstrual cycle of blood draw. ^2^ *p*-values were not statistically significant at the Bonferroni threshold. Abbreviations: Corr, correlation; GM, geometric mean; LCI, lower confidence interval; UCI, upper confidence interval; T1–3, tertiles 1–3; MBD, mammographic breast density; TDLU, terminal ductal lobular unit; MBD-V, mammographic breast density-volume; MBD-A, mammographic breast density-area; E2, unconjugated estradiol.

**Table 4:** Relationships of MBD and TDLU measures with circulating geometric mean concentrations of progesterone and progesterone metabolites (pmol/L) in follicular phase women.

		Pregnenolone	17a-Hydroxypregnenolone	Progesterone	17α-Hydroxyprogesterone	3αHP	5αP	20αHP	5αP/3αHP	E2	Progesterone/E2
	*N*	GM ^1^ (LCI-UCI)	GM ^1^ (LCI-UCI)	GM ^1^ (LCI-UCI)	GM ^1^ (LCI-UCI)	GM ^1^ (LCI-UCI)	GM ^1^ (LCI-UCI)	GM ^1^ (LCI-UCI)	GM ^1^ (LCI-UCI)	GM ^1^ (LCI-UCI)	GM ^1^ (LCI-UCI)
**MBD measures**											
**Percent MBD-V**											
T1	29	2319 (1860–2891)	3276 (2566–4182)	252 (174–367)	547 (429–697)	46 (38–56)	844 (629–1133)	218 (167–284)	18 (13–26)	140 (69–283)	2 (1–4)
T2	30	2835 (2325–3457)	3892 (3125–4848)	474 (339–664)	729 (586–906)	51 (43–61)	755 (579–983)	288 (227–366)	15 (11–20)	219 (112–429)	3 (1–7)
T3	29	2620 (2111–3250)	4032 (3176–5119)	297 (206–427)	585 (461–741)	39 (32–47)	687 (515–916)	216 (167–280)	18 (12–25)	153 (83–280)	2 (1–4)
P-trend ^2^		0.50	0.27	0.65	0.78	0.27	0.36	0.90	0.89	0.98	0.88
Corr ^1^ (rho, *p* ^2^)		(0.07, 0.50)	(0.14, 0.20)	(0.11, 0.31)	(0.05, 0.68)	(−0.12, 0.29)	(−0.09, 0.42)	(0.07, 0.51)	(−0.04, 0.74)	(−0.01, 0.92)	(−0.02, 0.91)
**Percent MBD-A**											
T1	29	2425 (1939–3031)	3295 (2575–4216)	374 (253–553)	560 (437–717)	46 (38–57)	977 (732–1303)	280 (213–366)	21 (15–30)	120 (58–245)	4 (2–9)
T2	30	2830 (2317–3457)	3831 (3072–4778)	320 (225–454)	697 (559–870)	47 (39–56)	773 (597–1001)	222 (174–282)	16 (12–23)	211 (118–380)	2 (1–4)
T3	29	2509 (2026–3108)	4074 (3217–5159)	301 (207–438)	597 (471–757)	43 (35–52)	579 (439–763)	221 (171–286)	14 (10–19)	167 (89–313)	2 (1–4)
P-trend ^2^		0.90	0.26	0.47	0.81	0.57	0.02	0.27	0.11	0.64	0.25
Corr ^1^ (rho, *p* ^2^)		(0.003, 0.98)	(0.14, 0.19)	(−0.02, 0.87)	(0.03, 0.79)	(−0.03, 0.77)	(−0.24, 0.03)	(0.01, 0.92)	(−0.16, 0.13)	(0.10, 0.46)	(−0.15, 0.29)
**Absolute MBD-V**											
T1	29	2338 (1899–2880)	3481 (2762–4388)	368 (255–530)	642 (509–810)	45 (37–54)	775 (587–1023)	248 (192–319)	17 (12–24)	117 (70–193)	3 (2–6)
T2	30	2762 (2264–3370)	3800 (3046–4739)	295 (208–418)	566 (453–706)	45 (37–54)	718 (550–936)	223 (175–284)	16 (11–22)	185 (97–351)	2 (1–4)
T3	29	2668 (2165–3288)	3889 (3084–4905)	333 (231–480)	646 (512–816)	46 (38–56)	789 (597–1042)	247 (192–319)	17 (12–24)	242 (129–451)	1 (1–3)
P-trend ^2^		0.39	0.52	0.71	0.97	0.83	0.93	0.99	0.96	0.07	0.11
Corr ^1^ (rho, *p* ^2^)		(0.10, 0.38)	(0.14, 0.19)	(0.04, 0.7)	(0.01, 0.93)	(0.04, 0.69)	(−0.03, 0.80)	(0.13, 0.22)	(−0.03, 0.81)	(0.26, 0.06)	(−0.23, 0.09)
**Absolute MBD-A**											
T1	29	2245 (1831–2753)	2966 (2378–3700)	306 (214–438)	545 (434–684)	52 (43–62)	740 (565–970)	242 (188–311)	14 (10–20)	144 (83–249)	2 (1–4)
T2	30	2854 (2337–3485)	4286 (3451–5323)	398 (280–566)	674 (539–842)	40 (33–47)	906 (696–1180)	252 (198–323)	23 (16–32)	139 (69–279)	4 (2–9)
T3	29	2687 (2198–3283)	4030 (3242–5010)	293 (206–417)	636 (509–796)	45 (38–54)	648 (497–845)	223 (174–285)	14 (10–20)	214 (120–383)	1 (1–3)
P-trend ^2^		0.24	0.06	0.84	0.36	0.30	0.47	0.64	0.99	0.33	0.30
Corr ^1^ (rho, *p* ^2^)		(0.12, 0.25)	(0.27, 0.01)	(0.04, 0.71)	(0.11, 0.33)	(−0.09, 0.40)	(−0.06, 0.55)	(0.07, 0.54)	(−0.01, 0.95)	(0.13, 0.35)	(−0.14, 0.31)
**TDLU involution measures**											
**TDLU count/100 mm^2^**											
T1	28	2408 (1954–2968)	3261 (2595–4098)	327 (229–465)	472 (379–588)	43 (35–52)	913 (691–1207)	275 (215–352)	21 (15–30)	79 (42–147)	4 (2–8)
T2	29	2939 (2404–3593)	4412 (3541–5496)	492 (350–692)	756 (612–934)	50 (42–60)	693 (530–906)	282 (223–358)	14 (10–19)	166 (94–293)	4 (2–8)
T3	29	2452 (1998–3010)	3652 (2919–4570)	227 (160–321)	675 (544–837)	43 (36–52)	719 (547–945)	180 (142–230)	17 (12–23)	273 (157–475)	1 (0–2)
P-trend ^2^		0.92	0.52	0.17	0.03	0.95	0.25	0.02	0.34	0.01	0.01
Corr ^1^ (rho, *p* ^2^)		(−0.02, 0.86)	(0.04, 0.72)	(−0.15, 0.17)	(0.19, 0.09)	(0.01, 0.95)	(−0.15, 0.18)	(−0.22, 0.04)	(−0.14, 0.20)	(0.38, 0.01)	(−0.36, 0.01)
**Median TDLU span, μ**											
T1	19	2473 (1920–3185)	3790 (2788–5153)	302 (184–496)	734 (547–986)	45 (36–57)	779 (548–1106)	214 (154–298)	17 (11–26)	186 (78–445)	3 (1–8)
T2	20	2707 (2138–3428)	4262 (3200–5677)	389 (245–618)	730 (555–961)	47 (38–58)	670 (483–930)	235 (172–320)	14 (10–21)	188 (99–355)	2 (1–6)
T3	20	2877 (2268–3650)	3726 (2792–4973)	356 (224–567)	698 (530–921)	48 (39–60)	705 (507–981)	249 (182–339)	15 (10–22)	267 (149–478)	1 (1–3)
P-trend ^2^		0.41	0.90	0.68	0.81	0.68	0.72	0.53	0.61	0.43	0.25
Corr ^1^ (rho, *p* ^2^)		(0.09, 0.50)	(−0.05, 0.70)	(0.11, 0.42)	(−0.02, 0.86)	(0.10, 0.47)	(−0.12, 0.38)	(0.21, 0.12)	(−0.10, 0.44)	(0.16, 0.34)	(−0.15, 0.37)
**Median acini count per TDLU**											
T1	18	2566 (1947–3382)	3940 (2809–5526)	297 (176–503)	778 (564–1074)	40 (31–51)	652 (445–954)	195 (137–277)	16 (10–26)	156 (66–371)	3 (1–9)
T2	19	3049 (2371–3921)	4011 (2948–5459)	557 (345–899)	802 (598–1076)	51 (41–65)	801 (566–1133)	305 (221–420)	16 (10–24)	274 (151–495)	2 (1–6)
T3	18	2638 (2045–3404)	3987 (2918–5447)	291 (179–472)	643 (478–866)	55 (44–70)	635 (447–903)	232 (168–321)	11 (8–18)	213 (114–398)	1 (0–3)
P-trend ^2^		0.99	0.97	0.75	0.36	0.08	0.82	0.65	0.25	0.74	0.23
Corr ^1^ (rho, *p* ^2^)		(−0.03, 0.80)	(0.02, 0.87)	(−0.11, 0.44)	(−0.10, 0.46)	(0.26, 0.06)	(−0.16, 0.26)	(0.08, 0.56)	(−0.22, 0.12)	(−0.02, 0.90)	(−0.24, 0.16)

^1^ Correlation adjusted for age and body mass index. ^2^ *p*-values were not statistically significant at the Bonferroni threshold. Abbreviations: Corr, correlation; GM, geometric mean; LCI, lower confidence interval; UCI, upper confidence interval; T1–3, tertiles 1–3; MBD, mammographic breast density; TDLU, terminal ductal lobular unit; MBD-V, mammographic breast density-volume; MBD-A, mammographic breast density-area; E2, unconjugated estradiol.

**Table 5:** Relationships of MBD and TDLU measures with circulating geometric mean concentrations of progesterone and progesterone metabolites (pmol/L) in postmenopausal women.

		Pregnenolone	17a-Hydroxypregnenolone	Progesterone	17α-Hydroxyprogesterone	3αHP	5αP	20αHP	5αP/3αHP	E2	Progesterone/E2
	*N*	GM ^1^ (LCI-UCI)	GM ^1^ (LCI-UCI)	GM ^1^ (LCI-UCI)	GM ^1^ (LCI-UCI)	GM ^1^ (LCI-UCI)	GM ^1^ (LCI-UCI)	GM ^1^ (LCI-UCI)	GM ^1^ (LCI-UCI)	GM ^1^ (LCI-UCI)	GM ^1^ (LCI-UCI)
**MBD measures**											
**Percent MBD-V**											
T1	34	1700 (1411–2047)	2929 (2382–3600)	107 (93–123)	355 (290–434)	51 (42–62)	832 (643–1076)	105 (91–120)	16 (12–23)	11 (8–16)	9 (6–13)
T2	35	1896 (1598–2249)	2805 (2321–3390)	124 (109–140)	376 (313–453)	48 (40–58)	877 (693–1111)	117 (103–133)	18 (13–25)	9 (6–13)	13 (9–19)
T3	34	1693 (1397–2052)	2869 (2318–3550)	120 (104–138)	417 (339–514)	44 (36–54)	655 (502–854)	115 (99–132)	15 (10–21)	8 (6–12)	15 (10–22)
P-trend ^2^		0.97	0.89	0.28	0.31	0.37	0.27	0.4	0.76	0.26	0.08
Corr ^1^ (rho, *p* ^2^)		(0.03, 0.80)	(0.06, 0.57)	(0.17, 0.10)	(0.14, 0.15)	(−0.07, 0.47)	(−0.12, 0.24)	(0.08, 0.42)	(−0.04, 0.67)	(−0.13, 0.23)	(0.19, 0.08)
**Percent MBD-A**											
T1	34	1513 (1264–1811)	2719 (2222–3327)	104 (91–119)	353 (289–430)	44 (36–54)	979 (761–1259)	103 (90–118)	22 (16–31)	12 (8–17)	9 (6–12)
T2	35	2037 (1723–2408)	2659 (2203–3209)	119 (105–135)	381 (316–459)	50 (42–61)	764 (604–966)	117 (103–133)	15 (11–21)	8 (6–12)	14 (9–20)
T3	34	1766 (1469–2124)	3265 (2654–4015)	128 (112–147)	415 (338–509)	48 (39–59)	642 (496–831)	117 (102–135)	13 (9–19)	8 (5–11)	16 (11–23)
P-trend ^2^		0.24	0.26	0.04	0.29	0.58	0.03	0.2	0.06	0.12	0.03
Corr ^1^ (rho, *p* ^2^)		(0.09, 0.38)	(0.05, 0.61)	(0.17, 0.09)	(0.08, 0.44)	(0.06, 0.56)	(−0.23, 0.02)	(0.13, 0.20)	(−0.21, 0.04)	(−0.18, 0.10)	(0.24, 0.02)
**Absolute MBD-V**											
T1	34	1593 (1339–1895)	2568 (2112–3122)	105 (92–120)	326 (269–394)	54 (44–65)	817 (638–1046)	108 (95–124)	15 (11–21)	8 (5–11)	14 (10–20)
T2	35	2098 (1773–2482)	3167 (2622–3826)	123 (109–140)	403 (335–484)	42 (35–51)	859 (676–1091)	113 (100–129)	20 (15–28)	10 (7–15)	11 (8–16)
T3	34	1627 (1371–1931)	2887 (2381–3500)	122 (108–139)	425 (352–512)	48 (39–57)	682 (535–871)	115 (100–131)	14 (10–20)	10 (7–15)	12 (8–17)
P-trend ^2^		0.92	0.43	0.12	0.06	0.42	0.31	0.57	0.78	0.21	0.54
Corr ^1^ (rho, *p* ^2^)		(−0.01, 0.91)	(0.07, 0.50)	(0.19, 0.06)	(0.20, 0.04)	(−0.08, 0.41)	(−0.11, 0.29)	(0.06, 0.54)	(−0.05, 0.62)	(0.16, 0.13)	(−0.09, 0.39)
**Absolute MBD-A**											
T1	34	1569 (1319–1868)	2710 (2228–3296)	107 (94–122)	378 (312–458)	44 (36–53)	987 (776–1255)	104 (91–119)	23 (16–31)	10 (7–15)	10 (7–15)
T2	35	2021 (1709–2391)	2886 (2389–3486)	116 (102–131)	350 (291–421)	51 (42–61)	753 (597–950)	117 (103–133)	15 (11–20)	8 (5–11)	15 (10–21)
T3	34	1716 (1445–2038)	3011 (2482–3652)	128 (112–145)	422 (350–510)	48 (40–58)	647 (510–820)	115 (101–131)	13 (10–18)	10 (7–14)	12 (9–18)
P-trend ^2^		0.52	0.46	0.07	0.42	0.49	0.02	0.29	0.03	0.85	0.48
Corr ^1^ (rho, *p* ^2^)		(0.04, 0.68)	(0.02, 0.83)	(0.15, 0.13)	(0.07, 0.47)	(0.07, 0.50)	(−0.25, 0.01)	(0.12, 0.24)	(−0.23, 0.02)	(−0.02, 0.84)	(0.07, 0.51)
**TDLU involution measures**											
**TDLU count/100 mm^2^**											
T1	37	1637 (1383–1937)	2886 (2393–3480)	113 (100–128)	114 (100–128)	44 (37–52)	989 (786–1245)	109 (96–123)	23 (17–31)	11 (8–16)	10 (7–14)
T2	32	1725 (1442–2064)	2989 (2448–3648)	113 (99–129)	367 (302–447)	57 (47–69)	666 (521–850)	103 (90–118)	12 (8–16)	7 (5–11)	14 (10–21)
T3	34	1946 (1628–2326)	2736 (2244–3335)	124 (109–142)	398 (328–484)	44 (36–53)	707 (555–902)	125 (109–142)	16 (12–22)	9 (7–13)	13 (10–19)
P-trend ^2^		0.18	0.72	0.33	0.76	0.89	0.05	0.18	0.13	0.42	0.22
Corr ^1^ (rho, *p* ^2^)		(0.15, 0.15)	(−0.01, 0.89)	(0.08, 0.44)	(0.04, 0.70)	(0.04, 0.69)	(−0.19, 0.06)	(0.07, 0.49)	(−0.12, 0.22)	(−0.07, 0.50)	(0.11, 0.32)
**Median TDLU span, μ**											
T1	22	1831 (1487–2253)	2980 (2372–3743)	121 (104–142)	406 (326–505)	46 (37–58)	807 (626–1041)	118 (101–138)	17 (12–24)	7 (5–11)	16 (10–26)
T2	22	1779 (1446–2189)	2503 (1994–3143)	109 (93–127)	322 (259–400)	49 (39–61)	648 (503–835)	110 (94–129)	13 (10–19)	7 (5–11)	15 (10–24)
T3	22	1981 (1608–2440)	3118 (2480–3920)	129 (110–151)	419 (337–521)	53 (42–67)	617 (478–797)	115 (99–135)	12 (8–16)	10 (6–17)	12 (7–19)
P-trend ^2^		0.60	0.79	0.62	0.85	0.42	0.15	0.83	0.1	0.32	0.34
Corr ^1^ (rho, *p* ^2^)		(0.02, 0.88)	(−0.01, 0.92)	(0.06, 0.63)	(−0.002, 0.99)	(0.10, 0.41)	(−0.18, 0.15)	(−0.01, 0.94)	(−0.24, 0.06)	(0.09, 0.49)	(−0.08, 0.56)
**Median acini count per TDLU**											
T1	23	1859 (1516–2279)	3077 (2456–3855)	124 (106–145)	376 (302–467)	48 (38–60)	631 (491–811)	124 (106–144)	13 (9–18)	8 (5–12)	16 (10–24)
T2	21	1902 (1530–2366)	2770 (2177–3525)	115 (97–136)	397 (314–501)	50 (39–63)	770 (589–1008)	106 (90–125)	15 (11–22)	6 (4–10)	18 (11–30)
T3	22	1826 (1482–2251)	2715 (2155–3420)	119 (101–139)	368 (294–460)	50 (40–63)	670 (518–867)	113 (97–132)	13 (10–19)	11 (7–17)	11 (7–17)
P-trend ^2^		0.91	0.44	0.68	0.9	0.81	0.73	0.42	0.92	0.34	0.28
Corr ^1^ (rho, *p* ^2^)		(−0.03, 0.80)	(−0.15, 0.23)	(−0.03, 0.80)	(−0.02, 0.85)	(0.03, 0.83)	(0.04, 0.75)	(−0.11, 0.37)	(0.01, 0.96)	(0.11, 0.42)	(−0.13, 0.35)

^1^ Correlation adjusted for age and body mass index. ^2^ *p*-values were not statistically significant at the Bonferroni threshold. Abbreviations: Corr, correlation; GM, geometric mean; LCI, lower confidence interval; UCI, upper confidence interval; T1–3, tertiles 1–3; MBD, mammographic breast density; TDLU, terminal ductal lobular unit; MBD-V, mammographic breast density-volume; MBD-A, mammographic breast density-area; E2, unconjugated estradiol.

## Supplementary Materials

The following are available online at https://www.mdpi.com/2077-0383/9/1/245/s1, Table S1: Age and body mass index-adjusted partial Spearman’s rank correlation coefficients (rho) for progesterone, progesterone metabolites, and unconjugated estradiol. Table S2: Distribution of progesterone, progesterone metabolites, unconjugated estradiol, MBD measures and TDLU measures, stratified by menopausal status and menstrual cycle phase, among BREAST Stamp Project participants with benign biopsy diagnoses. Table S3: Relationships of MBD and TDLU measures with circulating geometric mean concentrations of progesterone and progesterone metabolites (pmol/L) in luteal phase women with benign biopsy diagnoses. Table S4: Relationships of MBD and TDLU measures with circulating geometric mean concentrations of progesterone and progesterone metabolites (pmol/L) in follicular phase women with benign biopsy diagnoses. Table S5: Relationships of MBD and TDLU measures with circulating geometric mean concentrations of progesterone and progesterone metabolites (pmol/L) in postmenopausal women with benign biopsy diagnoses. Table S6: Relationships of MBD and TDLU measures with circulating geometric mean concentrations of progesterone and progesterone metabolites (pmol/L) in luteal phase women who reported regular menstrual cycle lengths (*N* = 47). Table S7: Relationships of MBD and TDLU measures with circulating geometric mean concentrations of progesterone and progesterone metabolites (pmol/L) in follicular phase women who reported regular menstrual cycle lengths (*N* = 59).
