# Supplementary material for: Relationship of Serum Progesterone and Progesterone Metabolites with Mammographic Breast Density and Terminal Ductal Lobular Unit Involution among Women Undergoing Diagnostic Breast Biopsy
Source: J Clin Med. 2020 Jan 17;9(1):245. doi: 10.3390/jcm9010245 (PMC7019918; doi:10.3390/jcm9010245)
Supplement: Supplementary file 1 [file jcm-09-00245-s001.pdf]

Supplemental Table S1. Age- and body mass index-adjusted partial Spearman's rank correlation coefficients (rho) for progesterone, progesterone metabolites and unconjugated estradiol.

**Luteal phase premenopausal women (N=65)**	Pregnenolone	17α-hydroxy pregnenolone	Progesterone	17α-hydroxy progesterone	3αHP	5αP	20αHP	E2
Pregnenolone	1.00							
17α-hydroxypregnenolone	0.62[1]	1.00						
Progesterone	0.30	-0.02	1.00					
17α-hydroxyprogesterone	0.39	0.12	0.56[1]	1.00				
3α-dihydroprogesterone (3αHP)	0.29	0.10	0.51[1]	0.17	1.00			
5α-dihydroprogesterone (5αP)	0.27	-0.02	0.72[1]	0.50[1]	0.45	1.00		
20α-Dihydroprogesterone (20αHP)	0.35	0.08	0.89[1]	0.40	0.57[1]	0.65[1]	1.00	
Unconjugated estradiol (E2)	0.34	0.12	0.05	0.64[1]	-0.14	0.16	0.03	1.00

**Follicular phase premenopausal women (N=88)**	Pregnenolone	17α-hydroxy pregnenolone	Progesterone	17α-hydroxy progesterone	3αHP	5αP	20αHP	E2
Pregnenolone	1.00							
17α-hydroxypregnenolone	0.50[1]	1.00						
Progesterone	0.37	0.24	1.00					
17α-hydroxyprogesterone	0.32	0.51[1]	0.57[1]	1.00				
3αHP	0.05	-0.01	0.09	0.11	1.00			
5αP	0.22	0.21	0.02	-0.06	-0.09	1.00		
20αHP	0.43[1]	0.22	0.76[1]	0.19	0.001	0.10	1.00	
E2	-0.01	0.12	0.09	0.48	0.19	0.39	-0.07	1.00

**Postmenopausal women (N=103)**	Pregnenolone	17α-hydroxy pregnenolone	Progesterone	17α-hydroxy progesterone	3αHP	5αP	20αHP	E2
Pregnenolone	1.00							
17α-hydroxypregnenolone	0.54[1]	1.00						
Progesterone	0.62[1]	0.47[1]	1.00					
17α-hydroxyprogesterone	0.37[1]	0.59[1]	0.69[1]	1.00				
3αHP	0.001	0.05	0.04	-0.02	1.00			
5αP	0.09	0.07	-0.01	-0.04	-0.15	1.00		
20αHP	0.53[1]	0.21	0.64[1]	0.14	0.04	0.12	1.00	
E2	-0.03	0.01	0.07	0.15	-0.04	0.10	0.04	1.00

[1] P-value < 0.0001

Supplemental Table S2. Distribution of progesterone, progesterone metabolites, unconjugated estradiol, MBD measures and TDLU measures, stratified by menopausal status and menstrual cycle phase, among BREAST Stamp Project participants with benign biopsy diagnoses.

	Luteal (N=58)		Follicular (N=76)		Postmenopausal (N=78)	
	Median	(IDR)	Median	(IDR)	Median	(IDR)
Hormones (pmol/L)						
Pregnenolone	3898	2310-7419	2780	1375-6017	1776	853-3291
17α-hydroxypregnenolone	3586	2353-8492	3497	1878-10482	2545	1508-6534
Progesterone	14333	1961-40437	278	122-1547	108	76.8-198.2
17α-hydroxyprogesterone	2112	786-3701	565	306-1675	325	195-909
3α-dihydroprogesterone (3αHP)	81.7	30.0-208.5	42.5	24.8-93.2	44.8	24.3-85.4
5α-dihydroprogesterone (5αP)	1969	713-5172	807	285-1821	832	283-2007
20α-Dihydroprogesterone (20αHP)	4095	540-11338	218	114-559	112	64.6-186.0
5αP/3αHP Ratio	23.4	8.3-54.8	17.7	5.0-52.8	17.5	4.9-72.5
Unconjugated estradiol (E2)	243	96-746	220	22.5-602.1	8.1	2.2-40.5
Progesterone/E2 Ratio	81.4	3.6-187.5	1.8	0.4-17.4	13.5	2.8-57.5

Abbreviation: IDR Interdecile Range

Supplemental Table S3. Relationships of MBD and TDLU measures with circulating geometric mean concentrations of progesterone and progesterone metabolites (pmol/L) in luteal phase women with benign biopsy diagnoses.

	Pregnenolone	17a-hydroxy pregnenolone	Progesterone	17a-hydroxy progesterone	3αHP	5αP	20αHP	5αP/3αHP	E2	Progesterone/ E2
	GM[1] (LCI-UCI)	GM[1] (LCI-UCI)	GM[1] (LCI-UCI)	GM[1] (LCI-UCI)	GM[1] (LCI-UCI)	GM[1] (LCI-UCI)	GM[1] (LCI-UCI)	GM[1] (LCI-UCI)	GM[1] (LCI-UCI)	GM[1] (LCI-UCI)
**Percent MBD-V**	3729 (2958-4701)	3872 (3002-4993)	8448 (4418-16153)	1689 (1293-2207)	91 (64-130)	1704 (1162-2501)	2784 (1590-4874)	19 (13-28)	226 (146-350)	46 (19-107)
T1	3705 (2977-4612)	3844 (3022-4889)	10057 (5449-18561)	1852 (1439-2385)	64 (46-90)	1505 (1047-2162)	3280 (1932-5569)	23 (16-34)	207 (138-311)	41 (19-92)
T2	4557 (3558-5838)	4059 (3093-5328)	11765 (5883-23527)	1881 (1414-2503)	84 (57-123)	2026 (1345-3052)	3535 (1943-6434)	24 (16-37)	280 (188-418)	46 (21-101)
T3	0.31	0.83	0.53	0.62	0.75	0.61	0.60	0.44	0.47	0.97
P-trend	(0.12, 0.41)	(-0.002, 0.98)	(0.11, 0.47)	(0.05, 0.71)	(0.02, 0.91)	(0.14, 0.34)	(0.12, 0.42)	(0.2, 0.17)	(0.05, 0.77)	(0.01, 0.97)
Correlation[1] (rho, p-value)										
**Percent MBD-A**	3424 (2823-4154)	3736 (2992-4666)	9576 (5593-16398)	1735 (1383-2178)	95 (70-130)	1550 (1104-2174)	3180 (2025-4993)	16 (12-23)	202 (140-291)	50 (25-102)
T1	3674 (3025-4463)	3605 (2882-4509)	5964 (3470-10251)	1532 (1219-1925)	60 (44-81)	1578 (1122-2220)	1864 (1183-2936)	26 (19-38)	221 (155-315)	29 (14-57)
T2	5275 (4226-6585)	4639 (3594-5988)	19867 (10711-36848)	2318 (1786-3008)	91 (64-130)	2238 (1517-3302)	6173 (3677-10364)	25 (17-37)	309 (212-449)	64 (31-132)
T3	0.01	0.26	0.16	0.16	0.68	0.19	0.15	0.10	0.13	0.68
P-trend	(0.32, 0.03)	(0.19, 0.19)	(0.21, 0.14)	(0.22, 0.13)	(-0.01, 0.97)	(0.21, 0.14)	(0.21, 0.14)	(0.31, 0.03)	(0.18, 0.28)	(0.07, 0.65)
Correlation[1] (rho, p-value)										
**Absolute MBD-V**	3679 (2961-4572)	4498 (3569-5669)	7099 (3935-12809)	1574 (1232-2009)	80 (57-112)	1396 (982-1983)	2391 (1442-3965)	18 (12-25)	215 (153-303)	33 (17-62)
T1	4134 (3340-5117)	3602 (2870-4521)	10117 (5666-18062)	1947 (1532-2475)	75 (54-104)	2040 (1445-2881)	2962 (1802-4869)	27 (19-39)	269 (189-382)	39 (20-74)
T2	4117 (3234-5242)	3718 (2875-4809)	14192 (7364-27351)	1911 (1456-2508)	85 (59-124)	1814 (1227-2681)	4709 (2683-8263)	21 (14-32)	238 (150-376)	94 (40-220)
T3	0.48	0.25	0.13	0.27	0.83	0.29	0.10	0.41	0.62	0.08
P-trend	(0.01, 0.95)	(-0.21, 0.14)	(0.14, 0.33)	(0.2, 0.17)	(0.06, 0.68)	(0.11, 0.43)	(0.18, 0.21)	(0.04, 0.76)	(0.13, 0.41)	(0.19, 0.23)
Correlation[1] (rho, p-value)										
**Absolute MBD-A**	3217 (2628-3939)	3866 (3075-4860)	7229 (4046-12915)	1664 (1304-2123)	79 (57-109)	1454 (1031-2051)	2427 (1469-4009)	18 (13-26)	186 (132-263)	46 (23-90)
T1	4527 (3718-5512)	4370 (3498-5459)	9852 (5603-17322)	1827 (1441-2315)	93 (68-128)	1638 (1172-2288)	3165 (1942-5156)	18 (13-25)	265 (187-377)	32 (16-64)
T2	4383 (3487-5510)	3482 (2689-4510)	15021 (7796-28941)	1956 (1485-2576)	66 (45-95)	2315 (1569-3415)	4446 (2521-7840)	35 (24-52)	293 (197-436)	65 (30-142)
T3	0.05	0.63	0.12	0.40	0.55	0.10	0.13	0.03	0.09	0.57
P-trend	(0.21, 0.15)	(-0.05, 0.75)	(0.19, 0.19)	(0.14, 0.33)	(-0.04, 0.78)	(0.22, 0.13)	(0.18, 0.21)	(0.27, 0.06)	(0.22, 0.18)	(0.11, 0.49)
Correlation[1] (rho, p-value)										
**TDLU Involution Measures**										
**TDLU count/100 mm^2**	3436 (2729-4325)	3524 (2740-4532)	10856 (5686-20727)	1804 (1380-2360)	95 (67-136)	1547 (1054-2272)	3682 (2130-6365)	16 (11-24)	190 (128-283)	52 (24-116)
T1	4065 (3311-4990)	3933 (3144-4921)	11189 (6289-19909)	1834 (1444-2329)	80 (58-110)	1988 (1412-2799)	3855 (2367-6277)	25 (17-35)	311 (225-431)	35 (18-67)
T2	4400 (3553-5449)	4300 (3404-5432)	8100 (4441-14771)	1764 (1375-2263)	67 (48-93)	1652 (1156-2361)	2253 (1355-3746)	25 (17-36)	211 (147-304)	52 (25-109)
T3	0.14	0.27	0.51	0.90	0.16	0.85	0.19	0.15	0.81	0.96
P-trend	(0.16, 0.26)	(0.17, 0.23)	(-0.16, 0.27)	(-0.08, 0.56)	(-0.15, 0.28)	(-0.04, 0.8)	(-0.22, 0.13)	(0.18, 0.22)	(0.04, 0.81)	(-0.05, 0.74)
Correlation[1] (rho, p-value)										
**Median TDLU span, μm**	4649 (3775-5727)	4362 (3354-5672)	12301 (7271-20810)	2023 (1644-2489)	79 (58-107)	1797 (1250-2583)	3846 (2400-6164)	23 (16-33)	329 (222-488)	37 (18-74)
T1	3609 (2908-4480)	3723 (2835-4889)	6816 (3952-11756)	1529 (1233-1895)	70 (51-96)	1848 (1268-2693)	2118 (1299-3453)	27 (18-39)	212 (141-321)	41 (20-85)
T2	4486 (3616-5567)	4149 (3161-5446)	11198 (6498-19297)	1796 (1449-2226)	80 (58-110)	1628 (1118-2371)	3200 (1964-5214)	20 (14-30)	217 (141-333)	53 (25-114)
T3	0.80	0.78	0.79	0.43	0.94	0.71	0.58	0.68	0.16	0.49
P-trend	(-0.03, 0.85)	(-0.05, 0.75)	(-0.05, 0.78)	(-0.12, 0.45)	(0.01, 0.97)	(-0.09, 0.57)	(-0.13, 0.43)	(-0.14, 0.4)	(-0.2, 0.27)	(0.1, 0.58)
Correlation[1] (rho, p-value)										
**Median acini count per TDLU**	4577 (3770-5557)	4405 (3487-5565)	12250 (7467-20097)	1985 (1624-2426)	82 (62-110)	2055 (1458-2896)	3684 (2350-5774)	25 (17-36)	331 (228-480)	38 (20-74)
T1	3433 (2786-4231)	3111 (2419-4001)	6189 (3632-10548)	1540 (1241-1911)	66 (48-90)	1493 (1032-2160)	2013 (1240-3266)	23 (15-34)	227 (145-354)	32 (15-70)
T2	4833 (3834-6094)	5002 (3783-6613)	12409 (6868-22420)	1793 (1411-2279)	80 (56-113)	1698 (1127-2559)	3500 (2046-5988)	21 (14-33)	188 (122-289)	64 (30-137)
T3	0.92	0.70	0.87	0.45	0.82	0.44	0.76	0.58	0.06	0.35
P-trend	(0.0002, 0.99)	(0.06, 0.74)	(-0.03, 0.87)	(-0.13, 0.43)	(-0.01, 0.95)	(-0.18, 0.27)	(-0.09, 0.58)	(-0.13, 0.42)	(-0.27, 0.13)	(0.08, 0.67)

[1]Adjusted for age, body mass index, and day in menstrual cycle of blood draw.

Abbreviations: GM, Geometric Mean; LCI, Lower Confidence Interval; UCI Upper Confidence Interval; T1-3, Tertiles 1-3; MBD Mammographic Breast Density; TDLU Terminal Ductal Lobular Unit, MBD-V Mammographic Breast Density-Volume, MBD-A Mammographic Breast Density-Area; E2 Unconjugated Estradiol; Bold values are P-value <0.05

Supplemental Table S4. Relationships of MBD and TDLU measures with circulating geometric mean concentrations of progesterone and progesterone metabolites (pmol/L) in follicular phase women with benign biopsy diagnoses.

	Pregnenolone	17a-hydroxy pregnenolone	Progesterone	17a-hydroxy progesterone	3αHP	5αP	20αHP	5αP/3αHP	E2	Progesterone/E2
	GM[1] (LCI-UCI)	GM[1] (LCI-UCI)	GM[1] (LCI-UCI)	GM[1] (LCI-UCI)	GM[1] (LCI-UCI)	GM[1] (LCI-UCI)	GM[1] (LCI-UCI)	GM[1] (LCI-UCI)	GM[1] (LCI-UCI)	GM[1] (LCI-UCI)
**Percent MBD-V**										
T1	2309 (1733-3077)	2722 (1987-3729)	201 (122-334)	479 (347-661)	52 (39-67)	707 (488-1025)	173 (125-239)	14 (9-22)	120 (62-234)	2 (1-4)
T2	3040 (2348-3937)	4126 (3107-5478)	718 (456-1130)	823 (615-1101)	57 (44-72)	887 (635-1240)	400 (299-535)	16 (10-24)	178 (94-336)	4 (2-11)
T3	3001 (2355-3826)	3942 (3021-5145)	281 (183-430)	627 (477-824)	37 (30-47)	682 (498-933)	220 (168-289)	18 (12-27)	173 (98-304)	2 (1-3)
P-trend	0.28	0.18	0.90	0.49	0.06	0.72	0.83	0.40	0.53	0.62
Correlation[1] ((rho, p-value))	(0.16, 0.24)	(0.2, 0.15)	(0.09, 0.51)	(0.08, 0.58)	(-0.25, 0.07)	(-0.01, 0.95)	(0.13, 0.34)	(0.07, 0.59)	(0.07, 0.64)	(-0.05, 0.73)
**Percent MBD-A**										
T1	2589 (1921-3491)	3172 (2268-4438)	356 (198-641)	558 (394-791)	50 (37-67)	887 (604-1304)	258 (177-378)	18 (11-29)	111 (56-220)	4 (1-10)
T2	3112 (2466-3928)	3691 (2841-4795)	388 (245-613)	697 (531-914)	51 (40-64)	754 (559-1018)	256 (190-344)	15 (10-22)	176 (103-299)	2 (1-4)
T3	2582 (2006-3322)	3716 (2799-4934)	265 (161-435)	604 (450-811)	41 (32-52)	634 (458-878)	217 (158-299)	15 (10-24)	180 (98-330)	1 (1-3)
P-trend	0.82	0.56	0.40	0.89	0.28	0.22	0.48	0.76	0.40	0.16
Correlation[1] ((rho, p-value))	(0.00008, 1.)	(0.14, 0.3)	(0.001, 0.99)	(-0.003, 0.98)	(-0.12, 0.39)	(-0.16, 0.26)	(0.13, 0.36)	(-0.03, 0.82)	(0.09, 0.54)	(-0.12, 0.42)
**Absolute MBD-V**										
T1	2512 (1991-3171)	3246 (2502-4210)	355 (224-563)	646 (492-849)	47 (37-59)	752 (555-1018)	236 (176-318)	16 (11-24)	126 (77-203)	3 (1-6)
T2	3031 (2305-3985)	3776 (2781-5128)	332 (193-571)	626 (454-863)	47 (36-62)	752 (526-1074)	244 (172-345)	16 (10-25)	152 (78-296)	2 (1-6)
T3	2863 (2263-3621)	3699 (2844-4811)	309 (194-492)	594 (451-783)	47 (37-59)	734 (540-997)	248 (184-335)	16 (11-23)	205 (117-359)	2 (1-3)
P-trend	0.46	0.50	0.69	0.68	0.99	0.92	0.82	0.93	0.21	0.26
Correlation[1] ((rho, p-value))	(0.14, 0.32)	(0.14, 0.3)	(0.06, 0.64)	(-0.03, 0.84)	(0.04, 0.76)	(-0.12, 0.39)	(0.26, 0.05)	(-0.08, 0.56)	(0.18, 0.24)	(-0.11, 0.46)
**Absolute MBD-A**										
T1	2369 (1877-2990)	2929 (2259-3798)	297 (187-473)	585 (443-773)	54 (43-69)	728 (534-991)	227 (168-306)	13 (9-20)	149 (89-249)	2 (1-4)
T2	3418 (2572-4541)	4255 (3099-5842)	476 (270-838)	710 (506-996)	41 (31-54)	833 (571-1214)	283 (196-409)	21 (13-33)	126 (63-251)	4 (2-11)
T3	2798 (2256-3471)	3760 (2956-4782)	294 (191-452)	606 (468-783)	45 (36-55)	712 (535-948)	234 (177-310)	16 (11-23)	179 (105-305)	1 (1-3)
P-trend	0.39	0.22	0.88	0.92	0.27	0.88	0.94	0.59	0.62	0.53
Correlation[1] ((rho, p-value))	(0.13, 0.34)	(0.22, 0.11)	(0.09, 0.53)	(0.03, 0.85)	(-0.12, 0.38)	(-0.08, 0.56)	(0.23, 0.1)	(0.01, 0.95)	(0.04, 0.77)	(-0.04, 0.82)
**TDLU Involution Measures**										
**TDLU count/100 mm^2**										
T1	2634 (1956-3547)	3032 (2180-4216)	266 (153-462)	411 (297-568)	41 (31-55)	744 (504-1097)	223 (155-320)	18 (11-29)	72 (41-127)	4 (2-9)
T2	3021 (2392-3816)	4096 (3162-5307)	547 (355-844)	775 (601-1000)	52 (41-65)	704 (518-955)	316 (238-420)	14 (9-20)	158 (95-262)	4 (2-8)
T3	2559 (2034-3220)	3357 (2603-4329)	234 (153-358)	643 (500-825)	46 (36-57)	786 (582-1061)	199 (150-263)	17 (12-25)	255 (156-417)	1 (0-2)
P-trend	0.75	0.83	0.43	0.11	0.78	0.77	0.40	0.95	0.002	**0.01**
Correlation[1] ((rho, p-value))	(-0.03, 0.82)	(0.06, 0.66)	(-0.1, 0.49)	(0.14, 0.31)	(-0.02, 0.88)	(0.06, 0.66)	(-0.11, 0.42)	(0.05, 0.74)	(0.3, 0.05)	(-0.28, 0.07)
**Median TDLU span, µm**										
T1	2625 (2008-3432)	3663 (2675-5016)	403 (219-744)	774 (542-1104)	51 (38-69)	820 (568-1185)	259 (176-384)	16 (10-25)	150 (68-333)	4 (1-13)
T2	2898 (2265-3708)	4212 (3155-5624)	443 (252-778)	731 (528-1014)	50 (38-66)	561 (400-787)	268 (187-383)	11 (7-17)	188 (107-332)	2 (1-6)
T3	2636 (2049-3393)	3032 (2255-4075)	263 (148-468)	593 (425-829)	43 (32-56)	925 (655-1308)	230 (159-332)	22 (14-33)	216 (126-369)	1 (1-3)
P-trend	0.99	0.38	0.32	0.30	0.38	0.58	0.65	0.33	0.47	0.16
Correlation[1] ((rho, p-value))	(0.12, 0.47)	(-0.02, 0.9)	(-0.16, 0.3)	(-0.15, 0.36)	(-0.13, 0.43)	(0.18, 0.25)	(0.08, 0.6)	(0.24, 0.13)	(0.04, 0.81)	(-0.07, 0.7)
**Median acini count per TDLU**										
T1	2331 (1764-3080)	3143 (2241-4407)	338 (176-651)	771 (521-1140)	41 (30-57)	669 (434-1032)	209 (136-321)	16 (9-28)	138 (61-312)	2 (1-9)
T2	3280 (2599-4141)	4033 (3040-5350)	596 (345-1031)	797 (575-1106)	53 (40-69)	796 (554-1144)	321 (224-459)	15 (10-24)	246 (145-418)	3 (1-6)
T3	2588 (2028-3301)	3612 (2689-4854)	232 (131-411)	555 (395-782)	51 (39-69)	743 (509-1085)	239 (164-347)	14 (9-23)	203 (117-351)	1 (0-3)
P-trend	0.84	0.67	0.26	0.18	0.41	0.80	0.86	0.77	0.65	0.27
Correlation[1] ((rho, p-value))	(-0.01, 0.95)	(0.09, 0.59)	(-0.28, 0.08)	(-0.16, 0.33)	(0.14, 0.38)	(-0.06, 0.7)	(0.04, 0.79)	(-0.11, 0.51)	(0.02, 0.9)	(-0.26, 0.16)

[1]Adjusted for age and body mass index..

Abbreviations: GM, Geometric Mean; LCI, Lower Confidence Interval; UCI Upper Confidence Interval; T1-3, Tertiles 1-3; MBD Mammographic Breast Density; TDLU Terminal Ductal Lobular Unit, MBD-V Mammographic Breast Density-Volume, MBD-A Mammographic Breast Density-Area; E2 Unconjugated Estradiol; Bold values are P-value <0.05

Supplemental Table S5. Relationships of MBD and TDLU measures with circulating geometric mean concentrations of progesterone and progesterone metabolites (pmol/L) in postmenopausal women with benign biopsy diagnoses.

	Pregnenolone	17a-hydroxy pregnenolone	Progesterone	17a-hydroxy progesterone	3αHP	5αP	20αHP	5αP/3αHP	E2	Progesterone/ E2
	GM[1] (LCI-UCI)	GM[1] (LCI-UCI)	GM[1] (LCI-UCI)	GM[1] (LCI-UCI)	GM[1] (LCI-UCI)	GM[1] (LCI-UCI)	GM[1] (LCI-UCI)	GM[1] (LCI-UCI)	GM[1] (LCI-UCI)	GM[1] (LCI-UCI)
**Percent MBD-V**										
T1	1777 (1429-2210)	3077 (2383-3975)	113 (96-132)	387 (307-488)	49 (39-60)	890 (664-1192)	108 (93-127)	18 (12-27)	10 (6-15)	11 (7-17)
T2	1976 (1611-2423)	2701 (2126-3432)	123 (106-142)	353 (284-438)	48 (39-58)	930 (707-1223)	120 (104-139)	20 (13-28)	10 (6-15)	12 (8-19)
T3	1558 (1244-1952)	2811 (2157-3663)	117 (100-138)	400 (315-509)	41 (33-51)	629 (465-851)	113 (96-133)	15 (10-23)	8 (5-12)	15 (10-23)
P-trend	0.50	0.63	0.73	0.89	0.29	0.16	0.70	0.63	0.60	0.37
Correlation[1] (rho, p-value)	(-0.07, 0.53)	(0.02, 0.89)	(0.1, 0.39)	(0.06, 0.6)	(-0.13, 0.26)	(-0.19, 0.11)	(0.04, 0.71)	(-0.07, 0.55)	(-0.09, 0.48)	(0.15, 0.22)
**Percent MBD-A**										
T1	1506 (1220-1859)	2640 (2052-3397)	111 (95-130)	356 (283-449)	45 (36-55)	880 (653-1184)	111 (95-130)	20 (13-29)	12 (8-18)	9 (6-14)
T2	2172 (1779-2651)	2607 (2053-3310)	117 (101-136)	368 (295-458)	46 (37-56)	807 (609-1069)	114 (98-132)	18 (12-26)	9 (6-13)	13 (9-20)
T3	1673 (1340-2089)	3396 (2602-4431)	125 (106-148)	417 (326-532)	46 (37-58)	733 (535-1003)	117 (99-138)	16 (10-24)	7 (5-11)	17 (11-26)
P-trend	0.41	0.25	0.36	0.41	0.87	0.44	0.67	0.51	0.12	**0.07**
Correlation[1] (rho, p-value)	(0.06, 0.61)	(0.05, 0.67)	(0.06, 0.58)	(0.06, 0.63)	(-0.01, 0.97)	(-0.1, 0.38)	(0.04, 0.76)	(-0.08, 0.47)	(-0.21, 0.09)	**(0.28, 0.02)**
**Absolute MBD-V**										
T1	1563 (1271-1922)	2523 (1971-3229)	102 (87-118)	311 (249-388)	47 (38-58)	850 (638-1133)	108 (92-126)	18 (12-27)	6 (4-9)	17 (11-26)
T2	2186 (1789-2670)	3305 (2604-4196)	128 (111-148)	430 (347-532)	43 (35-52)	928 (703-1225)	114 (98-132)	22 (15-32)	10 (6-15)	12 (8-18)
T3	1600 (1311-1954)	2792 (2200-3542)	124 (107-143)	405 (327-501)	47 (38-57)	665 (505-878)	119 (103-138)	14 (10-21)	12 (8-18)	11 (7-16)
P-trend	0.91	0.59	0.08	0.11	0.98	0.24	0.36	0.37	**0.03**	0.13
Correlation[1] (rho, p-value)	(-0.04, 0.74)	(0.04, 0.74)	**(0.23, 0.04)**	(0.22, 0.06)	(-0.01, 0.91)	(-0.15, 0.19)	(0.1, 0.39)	(-0.13, 0.25)	**(0.31, 0.01)**	(-0.22, 0.08)
**Absolute MBD-A**										
T1	1585 (1298-1935)	2670 (2102-3392)	114 (98-132)	387 (312-479)	43 (35-53)	901 (683-1189)	112 (97-130)	21 (14-30)	10 (7-15)	11 (8-17)
T2	2174 (1759-2686)	2935 (2277-3783)	114 (97-133)	326 (260-410)	45 (37-56)	830 (618-1113)	116 (99-136)	18 (12-27)	7 (4-11)	16 (10-25)
T3	1643 (1350-1999)	2988 (2361-3783)	124 (107-144)	422 (341-521)	48 (39-58)	703 (535-923)	113 (98-131)	15 (10-21)	10 (7-14)	12 (8-18)
P-trend	0.82	0.52	0.43	0.58	0.49	0.22	0.92	0.20	0.88	0.75
Correlation[1] (rho, p-value)	(0.01, 0.96)	(0.0009, 0.99)	(0.05, 0.66)	(0.05, 0.65)	(0.06, 0.6)	(-0.15, 0.2)	(0.02, 0.89)	(-0.16, 0.16)	(-0.02, 0.88)	(0.07, 0.56)
**TDLU Involution Measures**										
**TDLU count/100 mm^2**										
T1	1576 (1262-1967)	2799 (2157-3631)	113 (96-133)	357 (282-452)	42 (34-52)	1131 (846-1512)	112 (97-131)	27 (18-40)	13 (9-21)	8 (6-13)
T2	1728 (1393-2142)	3037 (2360-3908)	112 (96-131)	386 (307-486)	53 (43-65)	709 (536-939)	97 (83-112)	13 (9-20)	6 (4-10)	16 (10-25)
T3	1944 (1598-2364)	2772 (2203-3488)	125 (109-145)	391 (318-482)	43 (36-52)	688 (533-889)	130 (113-149)	16 (11-23)	9 (6-12)	15 (10-21)
P-trend	0.18	0.95	0.34	0.59	0.90	**0.02**	0.18	0.07	0.19	0.08
Correlation[1] (rho, p-value)	(0.18, 0.13)	(0.04, 0.71)	(0.13, 0.26)	(0.1, 0.4)	(0.06, 0.63)	**(-0.24, 0.04)**	(0.11, 0.37)	(-0.17, 0.15)	(-0.14, 0.27)	(0.22, 0.08)
**Median TDLU span, μm**										
T1	1894 (1511-2374)	3173 (2439-4128)	127 (107-150)	431 (341-544)	44 (35-55)	919 (711-1186)	123 (104-146)	21 (15-30)	7 (4-11)	18 (12-29)
T2	1847 (1475-2312)	2529 (1947-3285)	109 (92-130)	312 (248-394)	51 (41-64)	667 (517-861)	113 (95-133)	13 (9-18)	7 (5-11)	15 (10-24)
T3	1894 (1490-2406)	3006 (2275-3974)	125 (104-150)	426 (333-546)	45 (35-57)	556 (424-729)	108 (90-129)	12 (9-18)	10 (6-17)	12 (7-20)
P-trend	0.99	0.75	0.89	0.89	0.83	**0.01**	0.29	**0.04**	0.36	0.25
Correlation[1] (rho, p-value)	(-0.06, 0.66)	(-0.15, 0.28)	(-0.04, 0.79)	(-0.06, 0.65)	(0.03, 0.84)	**(-0.36, 0.01)**	(-0.11, 0.44)	**(-0.31, 0.03)**	(0.1, 0.49)	(-0.15, 0.32)
**Median acini count per TDLU**										
T1	1900 (1526-2364)	3289 (2548-4246)	127 (108-151)	412 (325-521)	47 (38-59)	690 (533-892)	125 (106-147)	15 (10-21)	7 (5-12)	17 (11-27)
T2	1982 (1550-2534)	2810 (2109-3743)	115 (95-139)	388 (298-506)	50 (39-63)	867 (649-1158)	108 (90-130)	17 (12-26)	5 (3-9)	20 (12-33)
T3	1761 (1396-2221)	2558 (1951-3353)	116 (97-139)	352 (274-452)	43 (34-54)	592 (451-778)	110 (92-130)	14 (10-20)	11 (7-17)	11 (7-18)
P-trend	0.65	0.18	0.46	0.37	0.55	0.47	0.27	0.86	0.33	0.22
Correlation[1] (rho, p-value)	(-0.09, 0.52)	**(-0.31, 0.03)**	(-0.1, 0.49)	(-0.16, 0.24)	(-0.06, 0.66)	(-0.11, 0.41)	(-0.16, 0.26)	(-0.02, 0.88)	(0.14, 0.35)	(-0.19, 0.19)

[1]Adjusted for age, body mass index, and day in menstrual cycle of blood draw.

Abbreviations: GM, Geometric Mean; LCI, Lower Confidence Interval; UCI Upper Confidence Interval; T1-3, Tertiles 1-3; MBD Mammographic Breast Density; TDLU Terminal Ductal Lobular Unit, MBD-V Mammographic Breast Density-Volume, MBD-A Mammographic Breast Density-Area; E2 Unconjugated Estradiol; Bold values are P-value <0.05

Supplemental Table S6. Relationships of MBD and TDLU measures with circulating geometric mean concentrations of progesterone and progesterone metabolites (pmol/L) in luteal phase women who reported regular menstrual cycle lengths (N=47).

		Pregnenolone	17a-hydroxy pregnenolone	Progesterone	17α-hydroxy progesterone	3αHP	5αP	20αHP	5αP/3αHP	E2	Progesterone/E2
	N	GM[1] (LCI-UCI)	GM[1] (LCI-UCI)	GM[1] (LCI-UCI)	GM[1] (LCI-UCI)	GM[1] (LCI-UCI)	GM[1] (LCI-UCI)	GM[1] (LCI-UCI)	GM[1] (LCI-UCI)	GM[1] (LCI-UCI)	GM[1] (LCI-UCI)
**MBD Measures**											
**Percent MBD-V**											
T1	15	3901 (3073-4952)	4271 (3236-5638)	9816 (5295-18199)	1690 (1256-2274)	83 (57-122)	1637 (1107-2419)	2876 (1636-5058)	20 (13-30)	247 (145-420)	40 (14-113)
T2	16	3632 (2959-4457)	3415 (2691-4334)	10177 (5991-17288)	1842 (1428-2377)	64 (46-90)	1852 (1324-2589)	3007 (1852-4880)	29 (20-41)	241 (158-370)	38 (17-87)
T3	16	4757 (3746-6041)	3929 (2975-5188)	19934 (10743-36988)	2278 (1692-3067)	110 (75-162)	2797 (1892-4136)	5891 (3348-10367)	25 (17-38)	336 (215-523)	64 (27-150)
P-trend[2]		0.28	0.75	0.14	0.20	0.33	0.08	0.11	0.47	0.36	0.47
Correlation[1] (rho, p-value[2])		(0.14, 0.41)	(-0.12, 0.45)	(0.32, 0.05)	(0.20, 0.22)	(0.19, 0.25)	(0.36, 0.02)	(0.35, 0.03)	(0.25, 0.13)	(0.13, 0.50)	(0.13, 0.48)
**Percent MBD-A**											
T1	15	3503 (2904-4226)	3767 (2935-4835)	11667 (7031-19359)	1926 (1503-2467)	91 (64-128)	1646 (1163-2329)	3345 (2174-5146)	18 (13-26)	226 (146-348)	55 (23-132)
T2	16	3479 (2906-4164)	3521 (2772-4472)	7214 (4440-11721)	1508 (1189-1913)	62 (45-87)	1852 (1328-2583)	2006 (1327-3032)	30 (21-42)	223 (149-334)	28 (13-63)
T3	16	5613 (4599-6852)	4342 (3331-5661)	24618 (14375-42159)	2494 (1916-3245)	104 (72-150)	2781 (1923-4022)	7989 (5055-12628)	27 (18-39)	389 (261-581)	64 (29-142)
P-trend[2]		0.004	0.49	0.11	0.26	0.70	0.06	0.04	0.14	0.09	0.80
Correlation[1] (rho, p-value[2])		(0.39, 0.01)	(0.09, 0.58)	(0.31, 0.06)	(0.21, 0.20)	(0.12, 0.46)	(0.34, 0.03)	(0.41, 0.01)	(0.34, 0.03)	(0.26, 0.17)	(0.08, 0.67)
**Absolute MBD-V**											
T1	15	3884 (3135-4812)	4174 (3276-5318)	9187 (5351-15772)	1779 (1369-2310)	73 (52-104)	1662 (1176-2349)	2768 (1685-4549)	23 (16-33)	266 (183-387)	33 (17-67)
T2	16	4079 (3213-5179)	3496 (2670-4579)	11774 (6450-21493)	2052 (1534-2746)	84 (57-124)	2222 (1512-3266)	3386 (1948-5888)	26 (18-39)	345 (213-559)	36 (15-88)
T3	16	4196 (3205-5493)	3866 (2851-5243)	18444 (9347-36392)	1924 (1385-2674)	94 (60-146)	2280 (1476-3522)	5436 (2911-10153)	24 (15-38)	222 (131-376)	104 (39-276)
P-trend[2]		0.65	0.54	0.14	0.60	0.38	0.21	0.13	0.72	0.83	0.11
Correlation[1] (rho, p-value[2])		(0.003, 0.98)	(-0.11, 0.49)	(0.19, 0.25)	(0.12, 0.46)	(0.27, 0.09)	(0.19, 0.25)	(0.18, 0.28)	(-0.05, 0.77)	(-0.07, 0.70)	(0.22, 0.25)
**Absolute MBD-A**											
T1	15	3354 (2711-4148)	3630 (2821-4672)	9254 (5263-16271)	1671 (1279-2184)	71 (49-102)	1534 (1078-2182)	2565 (1541-4271)	22 (15-31)	202 (131-312)	48 (21-111)
T2	16	4605 (3781-5607)	4331 (3428-5471)	11864 (7032-20016)	2235 (1744-2863)	94 (67-131)	2119 (1528-2938)	3573 (2228-5731)	23 (16-32)	356 (240-529)	28 (13-59)
T3	16	4229 (3380-5291)	3501 (2683-4566)	18410 (10155-33375)	1816 (1370-2408)	84 (57-124)	2610 (1800-3786)	5657 (3305-9680)	31 (21-46)	272 (181-408)	79 (36-173)
P-trend[2]		0.16	0.86	0.11	0.68	0.53	0.05	0.04	0.20	0.40	0.37
Correlation[1] (rho, p-value[2])		(0.19, 0.25)	(-0.05, 0.75)	(0.27, 0.10)	(0.08, 0.62)	(0.16, 0.33)	(0.33, 0.04)	(0.36, 0.02)	(0.20, 0.23)	(0.04, 0.85)	(0.23, 0.22)
**TDLU Involution Measures**											
**TDLU count/100 mm²**											
T1	15	3949 (3150-4950)	4024 (3109-5209)	11604 (6487-20759)	1966 (1495-2585)	82 (56-119)	1719 (1189-2484)	3399 (2004-5764)	21 (14-31)	263 (171-404)	45 (19-107)
T2	16	4187 (3363-5212)	3709 (2888-4763)	15104 (8598-26535)	2077 (1593-2708)	80 (56-115)	2184 (1529-3122)	4826 (2893-8050)	27 (19-39)	359 (248-521)	39 (18-83)
T3	16	3998 (3196-5002)	3801 (2943-4909)	10829 (6086-19270)	1711 (1304-2244)	87 (60-126)	2193 (1522-3159)	2961 (1754-4998)	25 (17-37)	201 (131-309)	61 (25-147)
P-trend[2]		0.94	0.77	0.86	0.49	0.82	0.38	0.71	0.52	0.43	0.63
Correlation[1] (rho, p-value[2])		(-0.07, 0.65)	(-0.04, 0.80)	(-0.01, 0.94)	(-0.15, 0.35)	(0.07, 0.67)	(0.14, 0.38)	(-0.05, 0.77)	(0.05, 0.77)	(-0.18, 0.34)	(0.16, 0.40)
**Median TDLU span, μ**											
T1	12	3958 (3124-5015)	3355 (2588-4350)	11150 (5954-20883)	1831 (1389-2414)	80 (55-117)	1689 (1141-2499)	3312 (1847-5937)	21 (14-32)	362 (217-603)	30 (11-79)
T2	12	4628 (3612-5930)	4416 (3364-5795)	17527 (9085-33815)	2255 (1689-3012)	97 (65-143)	3320 (2202-5004)	4638 (2517-8548)	34 (22-53)	285 (166-488)	66 (24-185)
T3	12	4044 (3188-5129)	3784 (2915-4911)	10049 (5351-18872)	1885 (1428-2488)	74 (51-109)	1675 (1130-2483)	2959 (1646-5318)	22 (15-34)	236 (146-383)	46 (18-117)
P-trend[2]		0.90	0.53	0.83	0.88	0.79	0.99	0.80	0.82	0.24	0.55
Correlation[1] (rho, p-value[2])		(0.03, 0.88)	(0.15, 0.45)	(-0.04, 0.84)	(-0.00005, 1.00)	(-0.05, 0.79)	(-0.04, 0.84)	(-0.07, 0.72)	(-0.06, 0.77)	(-0.21, 0.32)	(0.04, 0.85)

[1]Adjusted for age, body mass index, and day in menstrual cycle of blood draw.

[2] P-values were not statistically significant at Bonferroni threshold.

Abbreviations: GM, Geometric Mean; LCI, Lower Confidence Interval; UCI Upper Confidence Interval; T1-3, Tertiles 1-3; MBD Mammographic Breast Density; TDLU Terminal Ductal Lobular Unit, MBD-V Mammographic Breast Density-Volume, MBD-A Mammographic Breast Density-Area; E2 Unconjugated Estradiol.

Supplemental Table 7. Relationships of MBD and TDLU measures with circulating geometric mean concentrations of progesterone and progesterone metabolites (pmol/L) in follicular phase women who reported regular menstrual cycle lengths (N=59).

		Pregnenolone	17a-hydroxy pregnenolone	Progesterone	17α-hydroxy progesterone	3αHP	5αP	20αHP	5αP/3αHP	E2	Progesterone/E2
	N	GM[1] (LCI-UCI)	GM[1] (LCI-UCI)	GM[1] (LCI-UCI)	GM[1] (LCI-UCI)	GM[1] (LCI-UCI)	GM[1] (LCI-UCI)	GM[1] (LCI-UCI)	GM[1] (LCI-UCI)	GM[1] (LCI-UCI)	GM[1] (LCI-UCI)
**MBD Measures**											
**Percent MBD-V**											
T1	19	2336 (1472-3707)	2690 (1619-4471)	629 (280-1413)	639 (395-1034)	74 (51-107)	893 (512-1558)	414 (240-714)	12 (6-24)	167 (63-443)	5 (1-20)
T2	20	2930 (2123-4043)	4167 (2924-5937)	477 (271-839)	790 (566-1105)	46 (36-60)	790 (536-1164)	270 (184-394)	17 (11-28)	219 (106-454)	3 (1-9)
T3	20	2688 (1935-3734)	3672 (2558-5271)	226 (127-402)	551 (391-775)	33 (25-43)	708 (477-1053)	199 (135-293)	21 (13-35)	181 (97-335)	1 (1-3)
P-trend[2]		0.79	0.55	0.04	0.40	0.00	0.52	0.05	0.22	0.96	0.16
Correlation[1]											
(rho, p-value[2])		(0.05, 0.79)	(0.11, 0.53)	(-0.21, 0.22)	(-0.14, 0.44)	(-0.47, 0.01)	(-0.05, 0.77)	(-0.15, 0.41)	(0.22, 0.21)	(-0.07, 0.73)	(-0.21, 0.29)
**Percent MBD-A**											
T1	19	2346 (1592-3457)	2710 (1745-4209)	613 (296-1271)	707 (459-1089)	69 (49-96)	727 (446-1184)	399 (247-645)	11 (6-19)	155 (64-375)	6 (1-22)
T2	20	3336 (2466-4513)	4183 (2968-5894)	398 (226-702)	680 (486-953)	43 (33-56)	777 (531-1137)	249 (171-362)	18 (11-29)	235 (128-433)	2 (1-4)
T3	20	2355 (1700-3262)	3725 (2573-5393)	258 (140-477)	592 (412-851)	36 (27-47)	829 (550-1250)	210 (140-315)	23 (14-38)	165 (81-336)	2 (1-5)
P-trend[2]		0.83	0.39	0.09	0.53	0.01	0.70	0.07	0.07	0.97	0.28
Correlation[1]											
(rho, p-value[2])		(-0.05, 0.76)	(0.24, 0.18)	(-0.18, 0.31)	(-0.13, 0.46)	(-0.36, 0.04)	(0.11, 0.53)	(-0.14, 0.42)	(0.34, 0.05)	(-0.03, 0.89)	(-0.11, 0.58)
**Absolute MBD-V**											
T1	19	2379 (1734-3265)	2989 (2105-4244)	493 (273-888)	743 (530-1042)	51 (38-68)	672 (46- 983)	319 (216-472)	13 (8-21)	177 (98-321)	3 (1-8)
T2	20	2898 (2018-4160)	4018 (2691-6000)	370 (189-725)	620 (421-913)	45 (32-63)	848 (549-1310)	253 (162-396)	19 (11-32)	272 (135-546)	1 (0-4)
T3	20	2879 (2035-4073)	3945 (2686-5794)	298 (156-568)	594 (410-860)	41 (30-56)	861 (568-1307)	227 (148-348)	21 (13-36)	137 (66-284)	2 (1-7)
P-trend[2]		0.41	0.28	0.26	0.37	0.32	0.38	0.25	0.19	0.70	0.57
Correlation[1]											
(rho, p-value[2])		(0.18, 0.32)	(0.27, 0.12)	(-0.18, 0.31)	(-0.19, 0.27)	(-0.18, 0.31)	(0.16, 0.37)	(-0.04, 0.84)	(0.25, 0.15)	(-0.06, 0.78)	(-0.04, 0.86)
**Absolute MBD-A**											
T1	19	2623 (1930-3563)	2985 (2132-4178)	469 (264-833)	657 (473-914)	54 (41-71)	752 (519-1088)	323 (222-471)	14 (9-22)	195 (106-360)	3 (1-8)
T2	20	2294 (1450-3629)	3582 (2165-5926)	352 (149-831)	744 (454-1220)	42 (28-63)	672 (387-1169)	218 (124-384)	16 (8-32)	270 (112-653)	1 (0-5)
T3	20	2955 (2179-4008)	4267 (3054-5962)	328 (185-581)	614 (442-852)	40 (31-53)	870 (602-1256)	241 (166-351)	21 (14-34)	149 (79-279)	2 (1-7)
P-trend[2]		0.59	0.15	0.39	0.77	0.16	0.58	0.29	0.20	0.54	0.82
Correlation[1]											
(rho, p-value[2])		(0.13, 0.45)	(0.35, 0.05)	(-0.13, 0.48)	(-0.09, 0.63)	(-0.20, 0.25)	(0.14, 0.44)	(-0.10, 0.56)	(0.24, 0.18)	(-0.07, 0.73)	(-0.01, 0.96)
**TDLU Involution Measures**											
**TDLU count/100 mm^2**											
T1	20	2776 (1841-4186)	3770 (2379-5975)	288 (143-581)	517 (339-787)	42 (29-62)	826 (504-1355)	239 (148-387)	20 (10-36)	154 (71-335)	2 (1-6)
T2	19	2763 (1989-3837)	3464 (2397-5006)	689 (393-1208)	812 (580-1137)	49 (36-66)	755 (508-1121)	369 (251-542)	15 (9-25)	122 (65-229)	8 (3-18)
T3	20	2550 (1832-3551)	3535 (2439-5123)	274 (156-483)	635 (452-892)	45 (34-62)	774 (520-1153)	213 (144-314)	17 (10-28)	322 (169-614)	1 (0-2)
P-trend[2]		0.74	0.86	0.65	0.66	0.86	0.87	0.53	0.81	0.16	0.24
Correlation[1]											
(rho, p-value[2])		(-0.17, 0.35)	(-0.10, 0.57)	(-0.04, 0.81)	(0.03, 0.86)	(0.03, 0.89)	(0.01, 0.95)	(-0.08, 0.65)	(-0.03, 0.87)	(0.22, 0.28)	(-0.22, 0.29)
**Median TDLU span, μ**											
T1	13	2501 (1741-3593)	3100 (1995-4817)	655 (282-1523)	755 (481-1184)	55 (37-83)	780 (483-1262)	355 (198-638)	14 (7-27)	239 (78-729)	6 (1-30)
T2	13	2976 (2101-4215)	4482 (2935-6845)	501 (223-1126)	951 (617-1465)	50 (34-74)	665 (419-1054)	232 (132-407)	13 (7-24)	187 (81-430)	3 (1-10)
T3	13	2747 (1937-3894)	2983 (1951-4560)	257 (114-579)	580 (376-895)	40 (27-59)	1079 (679-1714)	231 (131-406)	27 (14-50)	235 (101-546)	1 (0-3)
P-trend[2]		0.75	0.84	0.13	0.39	0.27	0.33	0.34	0.16	0.94	0.10
Correlation[1]											
(rho, p-value[2])		(0.08, 0.73)	(-0.02, 0.92)	(-0.28, 0.19)	(-0.15, 0.48)	(-0.25, 0.23)	(0.21, 0.32)	(-0.04, 0.84)	(0.31, 0.14)	(0.004, 0.99)	(-0.31, 0.20)

[1]Adjusted for age and body mass index.

[2]P-values were not statistically significant at Bonferroni threshold.

Abbreviations: GM, Geometric Mean; LCI, Lower Confidence Interval; UCI Upper Confidence Interval; T1-3, Tertiles 1-3; MBD Mammographic Breast Density; TDLU Terminal Ductal Lobular Unit, MBD-V Mammographic Breast Density-Volume, MBD-A Mammographic Breast Density-Area; E2 Unconjugated Estradiol.
